# Mammalian TBX1 Preferentially Binds and Regulates Downstream Targets Via a Tandem T-site Repeat

**DOI:** 10.1371/journal.pone.0095151

**Published:** 2014-05-05

**Authors:** Raquel Castellanos, Qing Xie, Deyou Zheng, Ales Cvekl, Bernice E. Morrow

**Affiliations:** 1 Department of Genetics, Albert Einstein College of Medicine, Bronx, New York, United States of America; 2 Department of Ophthalmology, Albert Einstein College of Medicine, Bronx, New York, United States of America; 3 Department of Neurology, Albert Einstein College of Medicine, Bronx, New York, United States of America; Karlsruhe Institute of Technology, Germany

## Abstract

Haploinsufficiency or mutation of *TBX1* is largely responsible for the etiology of physical malformations in individuals with velo-cardio-facial/DiGeorge syndrome (VCFS/DGS/22q11.2 deletion syndrome). *TBX1* encodes a transcription factor protein that contains an evolutionarily conserved DNA binding domain termed the T-box that is shared with other family members. All T-box proteins, examined so far, bind to similar but not identical consensus DNA sequences, indicating that they have specific binding preferences. To identify the TBX1 specific consensus sequence, Systematic Evolution of Ligands by Exponential Enrichment (SELEX) was performed. In contrast to other TBX family members recognizing palindrome sequences, we found that TBX1 preferentially binds to a tandem repeat of 5′-AGGTGTGAAGGTGTGA-3′. We also identified a second consensus sequence comprised of a tandem repeat with a degenerated downstream site. We show that three known human disease-causing *TBX1* missense mutations (F148Y, H194Q and G310S) do not alter nuclear localization, or disrupt binding to the tandem repeat consensus sequences, but they reduce transcriptional activity in cell culture reporter assays. To identify *Tbx1*-downstream genes, we performed an *in silico* genome wide analysis of potential *cis*-acting elements in DNA and found strong enrichment of genes required for developmental processes and transcriptional regulation. We found that TBX1 binds to 19 different loci *in vitro*, which may correspond to putative *cis*-acting binding sites. *In situ* hybridization coupled with luciferase gene reporter assays on three gene loci, *Fgf8, Bmper, Otog-MyoD*, show that these motifs are directly regulated by TBX1 *in vitro*. Collectively, the present studies establish new insights into molecular aspects of TBX1 binding to DNA. This work lays the groundwork for future *in vivo* studies, including chromatin immunoprecipitation followed by next generation sequencing (ChIP-Seq) to further elucidate the molecular pathogenesis of VCFS/DGS.

## Introduction

T-box genes encode a large family of transcription factors that are required during embryonic development. *Brachyury*, the founding member of this family was first identified due to the presence of a short tail phenotype found in heterozygous mice and lack of axial development with early lethality in null mutant embryos [1]–[3]. Brachyury has an evolutionarily conserved DNA binding domain, termed the T-box, and can regulate transcription of a reporter gene in cell culture [4], [5]. Since the original discovery of *Brachyury*, nineteen different T-box genes have been identified and are evolutionarily conserved from flies to humans [6]–[8]. Most T-box genes are dispersed on different chromosomes. They are classified based upon sequence homology to each other and are members of five different subfamilies [6]–[8]. As for Brachyury, most T-box transcription factors are required for embryonic development and many are sensitive to altered gene dosage for biological function. The T-box family has received notoriety as mutations have been identified in the etiology of several congenital malformation disorders. For example, mutations in *TBX3* lead to Ulnar Mammary Syndrome, and mutations in *TBX5* cause Holt-Oram Syndrome, both of these presenting disease specific limb and heart defects [9], [10] among others [11].

All T-box family members share an evolutionarily conserved, DNA binding domain comprising approximately 180 amino acids. The Brachyury protein binds as a homodimer to a palindrome of two AGGTGTGA “half-sites” [4]. Brachyury can also bind as a monomer to a single half-site, but with 20 fold lower binding affinity [12]. Molecular biological methods have been used to identify the consensus sequence for other T-box proteins and most can bind to the Brachyury palindrome or half-site [4], [12], [13], but they have their own preferential binding site, as in the case of TBX5, TBX6, TBX15 and TBX18 [14]–[16]. Among other T-box proteins tested, Brachyury, TBX15 and Eomes can bind to a direct repeat [16]–[18].

The *TBX1* gene encodes a T-box transcription factor that maps to the 22q11.2 region, which is hemizygously deleted in individuals with velo-cardio-facial syndrome and DiGeorge syndrome (VCFS/DGS; MIM #: 192430/188400). Since most have a typical 3 million base pair deletion, it is also referred to as 22q11.2 deletion syndrome (22q11DS). Historically, *TBX1* was found to bind to the palindromic T-site, but unlike for other transcription factors, it did not significantly activate nor repress transcription of a reporter construct [12]. Heterozygous mutations in *TBX1* have been reported in rare non-deleted patients with related physical defects to that of VCFS/DGS. It is believed that these are loss of function mutations resulting in haploinsufficiency [19]–[21].

As for other T-box genes, *Tbx1* is required in a dose dependent manner for normal mouse embryogenesis [22]–[24]. Inactivation of *Tbx1* results in neonatal lethality and embryos have a cleft palate, abnormal inner ears, absent thymus, parathyroid glands and persistent truncus arteriosus [22]–[25]. Unbiased gene profiling on RNA from microdissected tissues [25]–[28] and candidate gene approaches, based upon similar knockout phenotypes, have been undertaken to identify downstream genes of *Tbx1* [25], [26], [28]. Among hundreds of genes identified, only eight direct downstream transcriptional target genes were found, including *Fgf8*, *Fgf10*, *Pitx2*, *Chd7*, *Vefr3*, *Eya1* and *Wnt5a* [29]–[36].

To expand the repertoire of direct transcriptional downstream target genes, we performed Systematic Evolution of Ligands by Exponential Enrichment (SELEX) to identify the mouse TBX1 consensus site [4], [37]–[40]. We found that TBX1 binds to two different consensus sequences, one that is a perfect tandem repeat of the Brachyury half-site and the other that is an imperfect tandem repeat. TBX1 can activate transcription of these novel sites in luciferase reporter assays in cell culture. Using these new consensus TBX1 sites, we found that the TBX1 mutations previously reported alter transcriptional activity. Next, we wanted to use the new consensus sequences to identify potential downstream transcriptional target genes. After performing an *in silico* genome wide search for these motifs, we tested 30 and validated 11 putative direct binding sites, including sites in the *Fgf8*, *Bmper* and *Otog-MyoD* genomic loci. These and others are strong candidates to be pursued as direct downstream targets in future by *in vivo* functional experiments.

## Materials and Methods

### Ethics Statement

Animal studies were carried out in strict accordance with the recommendations in the Guide for the Care and Use of Laboratory Animals of the National Institutes of Health. The protocol was approved by the Albert Einstein College of Medicine Animal Institute Committee (Protocol Number: 2013–0405; Protocol Name: Mouse Models of 22q11 Rearrangement Disorders). All embryo dissections were conducted after euthanizing mice by direct inhalation with CO_2_.

### Recombinant GST-TBX1 Fusion Protein

The T-box region (amino acids 90–303) of mouse *Tbx1* was PCR amplified from cDNA with the flanking restriction enzyme sites of EcoRI and XhoI. These sites were used to subclone the DNA fragment into the bacterial expression vector, pGEX4t3 (GE Healthcare), to generate a GST-TBX1 fusion protein. The vector was transformed into BL21(DE3)LysS competent cells (Stratagene) and grown on LB ampicillin agar plates. Colonies were picked and grown in 2XYT media, 10 mg/ml Amp, 1 M MgCl_2_ and 20% glucose. Cultures were grown at 29°C and protein expression was induced by the addition of 100 mM IPTG (isopropyl-beta-D-thiogalactopyranoside). After induction with IPTG, protein was detected via Coomassie blue staining and the fusion protein was subsequently purified with glutathione Sepharose 4B beads (GE Healthcare) and detected via western blot. The same protocol was followed when inducing the F148Y, H194Q and G310S mutated TBX1 proteins.

### *In vitro* Selection (SELEX)

A 76-mer single-stranded library of oligonucleotides 5′-GTAACGTCGAGACGGAATTCGCGGCCGCN_18_CTCGAGGATCCGTGCTCAGTCCCTATCG-3′, where a random 18-mer sequence flanked by two 28-mer flanking fixed sequences used for sequencing, was synthesized by Fisher Scientific (HPLC purification) as previously described [41]. The second strands were generated using Klenow enzyme (NEB) at 25°C for 3 hrs with the primer 5′-CGATAGGGACTGAGCACGGATCCCT-3′. The dsDNA samples were separated on a 4.5% UltraPure Agarose 1000 gel (Invitrogen) and purified by Qiaquick Gel Extraction Kit (Qiagen). PCR was performed to amplify the dsDNA products. After six rounds of selection with recombinant GST-TBX1 protein, the PCR products from round 0 (original dsDNA randomers), two, four and six were labeled with [α-^32^P] dCTP (PerkinElmer, Cat# NEG513H250UC) by Taq DNA polymerase. Oligonucleotides from each round were captured using glutathione Sepharose beads (GE Healthcare). Each labeled round of oligonucleotides was tested via EMSA to determine the round with the highest enrichment. Amino acids 90-303 were digested from a plasmid containing full length *Tbx1* cDNA (*Tbx1*-pCDNA3.1) and subcloned into the pGEX 4t3 vector for bacterial induction (Figure S1a). Protein induction by IPTG was detected via Coomassie blue staining as well as by western blot analysis. The SELEX procedure was carried out for six rounds, incrementing the pool of oligonucleotides with the highest binding affinity with each subsequent round. The PCR products from round six were cloned into pSC-A vector for sequencing. In total, 60 colonies were picked, plasmid DNA was extracted and subjected to Sanger sequencing (Einstein Genomics Core Lab). The sequences were aligned using the WebLogo program (http://weblogo.berkeley.edu) and two motifs were generated.

### Western Blot

Proteins that were induced by IPTG and visualized with Coomassie staining were also tested via western blot with specific antibodies. Proteins were denatured with 6x Laemmli loading buffer to 95°C for 5 minutes. Samples were then loaded onto a 10% acrylamide/bisacrylamide gel for 1 hour at 120 volts and subsequently transferred onto a PVDF membrane (BioRad). Antibodies used were: 1°- rabbit polyclonal α mouse Tbx1 1∶500 (Zymed); rabbit polyclonal α GST 1∶500 (Abcam); 2°- ECL donkey anti-rabbit IgG, horseradish peroxidase linked whole antibody 1∶10,000 (Amersham Biosciences).

### Electrophoretic Mobility Shift Assays (EMSA)

Oligonucleotides were end-labeled with [γ-^32^P] dATP and T4 Polynucleotide Kinase (NEB), and purified with G-50 Sephadex columns (Roche). EMSA reactions were carried out in 12.5 ul total volumes. GST-TBX1 (5–25 µg) was pre-incubated with 1 µg/ul poly dI-dC, 100x unlabeled self-competitor in A_100_ buffer (0.5 M HEPES, 50% glycerol, 0.5 M EDTA, 1 M MgCl_2_, 1 M KCl, 0.1M DTT, H_2_O, proteinase k) for ten minutes at room temperature as previously described [42]. Double stranded [γ-^32^P] dATP labeled oligonucleotide was added together with 20% Ficoll, and 10X EMSA buffer (0.5M HEPES, 1 M MgCl_2_, 1 M KCl, 0.1 MDTT) and incubated for an additional ten minutes at room temperature. Reactions were then loaded onto a 5% acrylamide gel and the protein-DNA complexes were electrophoresed at 200 volts for 80 minutes.

### Reporter Constructs

Two oligonucleotides containing six copies of the tandem repeat-AGGTGTGAAGGTGTGA (6x TR) and half site partial site – AGGTGTGATCGCGTCAT (6x ½ SPS) in tandem respectively, were generated synthetically from Genscript. The 6x tandem oligonucleotides were digested with XhoI and NheI and subcloned into a pGL3-promoter vector (Promega) [43]. Each tandem repeat binding site had a spacer of two random nucleotides (6xTR-pGL3p and 6x½SPS-pGL3p). After testing a concentration gradient of full length *Tbx1*-pCDNA3.1, we determined that 100 ng of this plasmid led to the highest fold change (Fc-27) when compared to the control experiment where the empty activating vector (Empty pCDNA3.1) was co-transfected with the reporter. Mutated binding sites were generated synthetically from Genscript. The AGGTGTGA sequence was mutated to AATTTTGA (Mutated 6xTR-pGL3p and Mutated 6x½SPS-pGL3p) [31]. In addition mutations at positions P8 A→T, P11 G→C and P13 G→T in the ½SPS, were also generated (P8 6x½SPS-pGL3p; P11 6x½SPS-pGL3p; P13 6x½SPS-pGL3p).

### Luciferase Assays

Jeg3 cells (ATCC) were co-transfected with 25 ng of one reporter construct (6x TR-pGL3p; 6x½SPS-pGL3p; Mut. 6xTR-pGL3p; Mut. 6x½SPS-pGL3p; P8 6x½SPS-pGL3p; P11 6x½SPS-pGL3p; and P13 6x½SPS-pGL3p), 25-200 ng of activating construct (*Tbx1*-pcDNA3.1, F148Y *Tbx1*-pCDNA3.1, H194Q *Tbx1*-pcDNA3.1, G310S *Tbx1*-pcDNA3.1) and 5 ng of the internal control pRL TK vector (Promega). Cells were grown in 10% FBS in DMEM (Invitrogen), trypsinized and plated onto 48 well plates. The following day, cells were transected with the above constructs together with Lipofectamine LTX (Invitrogen), in MEM (Invitrogen). Four hours later, the media was changed to 10% FBS in DMEM. Luciferase assay readings were carried out 48 hours later using the Dual Luciferase Reporter Assay System (Promega). All data are presented as means ±SD; n≥3. P-values were determined using the Student's t-test.

### Immunofluorescence

Jeg3 cells were grown in 10% FBS in DMEM, trypsinized and plated onto 6-well plates. Cells were transfected 24 hours later with 500 ng of *Tbx1*-pcDNA3.1, F148Y *Tbx1*-pCDNA3.1, H194Q *Tbx1*-pcDNA3.1, or G310S *Tbx1*-pcDNA3.1, and lipofectamine LTX (Invitrogen). Cells were fixed with 4% paraformaldehyde and 4% sucrose 48 hours later, for 15 minutes at room temperature. Cells were permeabilized with 0.3% Triton X-100 and blocked with 10% BSA/PBS for 30 minutes at 37°C. Cells were then incubated with rabbit polyclonal α mouse TBX1 1∶500 (Zymed) for two hours at 37°C and then with Alexa Fluor 488 goat α mouse IgG (Invitrogen) and DAPI stain (1∶500) for one hour at 37°C.

### Site Directed Mutagenesis

The Quick Change Lightning Site-Directed Mutagenesis Kit (Agilent Technologies) was used to generate mutations in TBX1. Full length *Tbx1*-pCDNA3.1 was used as a template for the PCR reaction. Primers were designed with the nucleotide changes: F148Y Sense- 5′-CCCCACGTTCCAAGTGAAGCTTATGGAATGGATCC-3′; H194Q Sense- 5′-CTGGCCGAGTACAGTACCACCCGGACT-3′; G310 Sense- 5′-AACCACCGGCCCAGTGCGCTGCCGCTC-3′. After DpnI digestion, plasmids were transformed into XL10-Cold Ultracompetent cells and plated overnight on LB-ampicillin plates, per instructions supplied with kit. Colonies were picked and grown in liquid culture overnight. Plasmid DNA was isolated using the Qiagen mini-prep kit and subjected to Sanger sequencing.

### Bioinformatic Analysis

An in-house bioinformatic program was created to search the mouse genome for the consensus T-box motif, AGGTG(T/C)(G/T)A, identified by the SELEX experiment. These sites were then compared to a list of the most conserved elements produced by the phastCons database based on whole-genome alignment of placental mammals [44] from the UCSC genome browser (http://genome.ucsc.edu). Motifs were then assigned to the nearest RefSeq genes and were then grouped based on conservation and distance to transcriptional start sites (TSS) defined as −100 kb to +1 kb and −1 kb to +100 kb. Gene ontology software tools, GREAT (Genomic Regions Enrichment of Annotations Tool- http://bejerano.stanford.edu/great/public/html/index.php) and DAVID (http://david.abcc.ncifcrf.gov), were used to generate functional groups of genes harboring the motifs, by inputting the chromosomal positions of the putative TBX1 motifs.

### Whole-Mount RNA *In Situ* Hybridization

Embryos were fixed in 4% paraformaldehyde overnight at 4°C. The embryos were then serially dehydrated to 100% methanol and stored at −20°C. On day 1 of the protocol, the embryos were rehydrated to 1xPBS/.01%Tween-20 and the *in situ* hybridization assay was carried out as previously described (Franco et al., 2001). Anti-sense digoxigenin labeled RNA probes to *Tbx1* and *Fgf8* [45] were generated from plasmids via standard methods. The *Bmper* probe was generated from templates amplified from E10.5 mouse cDNA using the following primers: Fwd (5′-AGTCCTTGACTTGGCTTATC-3′; Rev (5′-GCACTTGGACATTATACTTGC-3′). Each RNA template was created by PCR with a T3 RNA polymerase binding site at the 5′ end and a T7 RNA polymerase binding site at the 3′ end. Embryos were dissected at E9.5 and E10.5. Mice were maintained in a 12 hour dark/12 hour light cycle in compliance with the Albert Einstein College of Medicine of Yeshiva University Institutional Animal Care and Use Committee (IACUC).

## Results

### SELEX Identifies the Optimal Binding Sites of GST-TBX1

**Figure 1:**
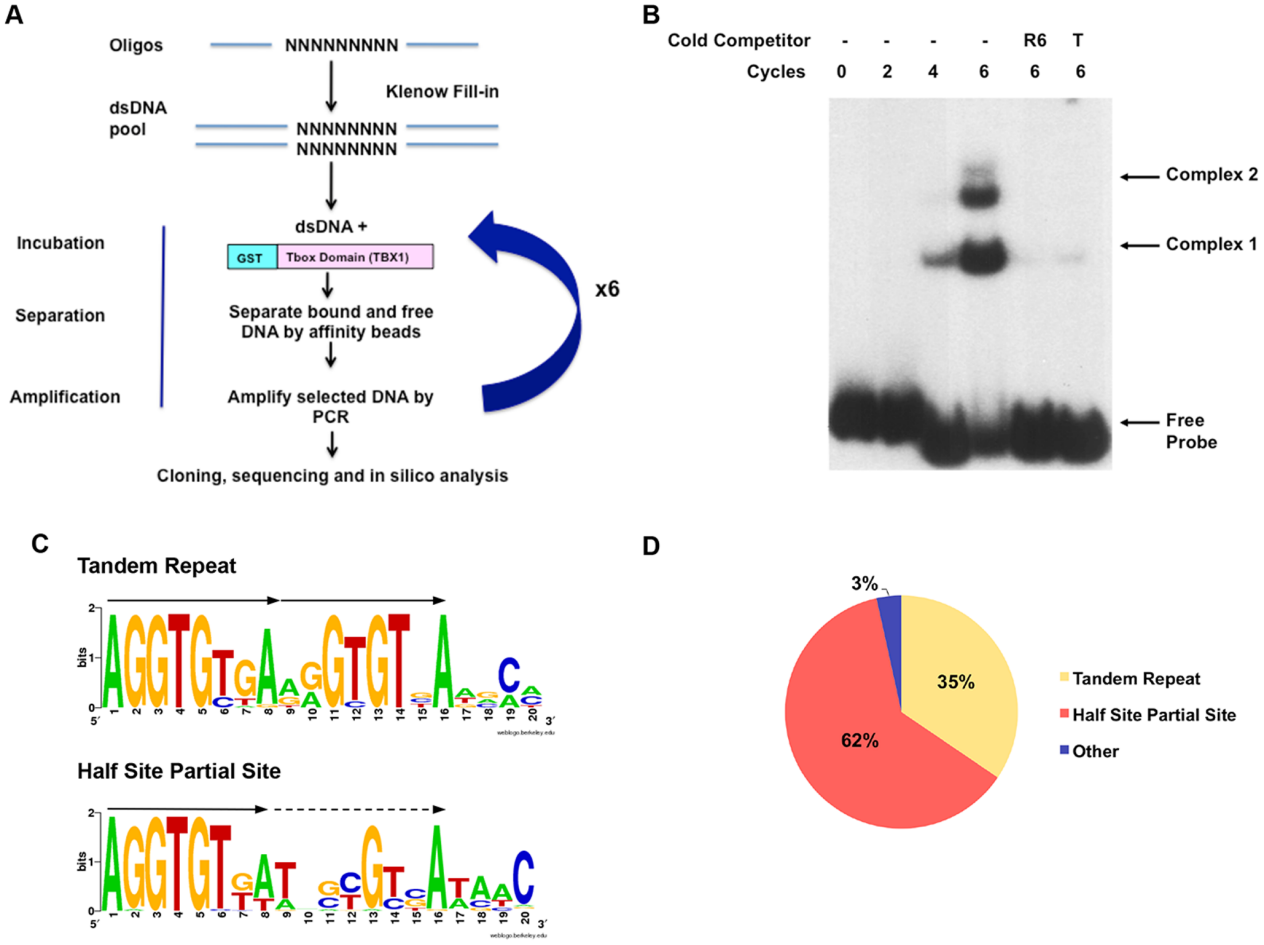
SELEX-Selection of specific oligonucleotides bound to GST-TBX1. A: A pipeline illustrating the SELEX method is shown. The dsDNA was generated by PCR of the selected oligonucleotides at each found and incubated with GST-TBX1. A total of 6 rounds of selection was performed. B: EMSA was used to detect specific GST-TBX1 and [α-32P]dCTP labeled DNA complexes at 0, 2, 4 and 6 rounds of selection, with or without cold competitor (R6, cold PCR products from round 6; T, ds DNA harboring the published Brachyury half site). C: Sequence alignment shows that the optimal DNA binding motif for TBX1 is AGGTGT(G/T)(A/T) followed by two repeated similar motifs termed the Tandem Repeat (TR) and Half Site Partial Site as shown (½SPS). D: Distribution of sequences with different consensus binding motifs within the pool of oligonucleotides after 6 rounds of selection (total number  = 60).

To determine the optimal binding site of mouse TBX1, an *in vitro* selection method termed Systematic Evolution of Ligands by Exponential Enrichment (SELEX) was performed. We created a GST-TBX1 fusion protein containing the T-box DNA binding domain [12] and ten amino acids on either side (90–303) (Fig. S1). The GST-TBX1 protein was able to bind to the Brachyury palindromic sequence as determined by electrophoretic mobility shift assay (EMSA) (Fig. S1). The validated GST-TBX1 protein was then subjected to the *in vitro* SELEX selection method and after six rounds of selection, we identified a clearly distinguishable protein-DNA complex (Fig. 1A). Both protein-DNA complexes were specific because binding of the radiolabeled oligonucleotides was competed with non-radiolabeled oligonucleotides obtained from round six of selection (Fig. 1B). We found two different sized protein-DNA complexes in gel assays, suggesting that TBX1 binds in two conformations (Fig. 1B). A total of 60 separate bacterial clones containing enriched oligonucleotides were sequenced to obtain a consensus sequence. Among them, 55 sequences were selected and aligned to generate two 16 bp DNA consensus sequences, containing one or two repeated GTGT “core” motifs in a tandem orientation (Figs. 1C,D). The GTGT core motif is part of the consensus binding site for T-box protein family members, indicating overlap between the TBX1 motif and that of other members. One consensus closely resembles the Brachyury half-site of AGGTGTGA. We termed this motif as the TBX1-TR (Fig. 1C). The second consensus sequence is also a tandem repeat, however, the 3′ site is comprised from a degenerate sequence with only two highly conserved positions (13 and 16, Fig. 1C). We termed the second consensus sequence, TBX1-½SPS (half site partial site) (Fig. 1C). In total, 62% of 55 sequences closely resembled the TR site and 34% had the ½SPS site consensus (Fig. 1D).

### Two Identified Motifs are Specifically Bound by GST-TBX1

**Figure 2:**
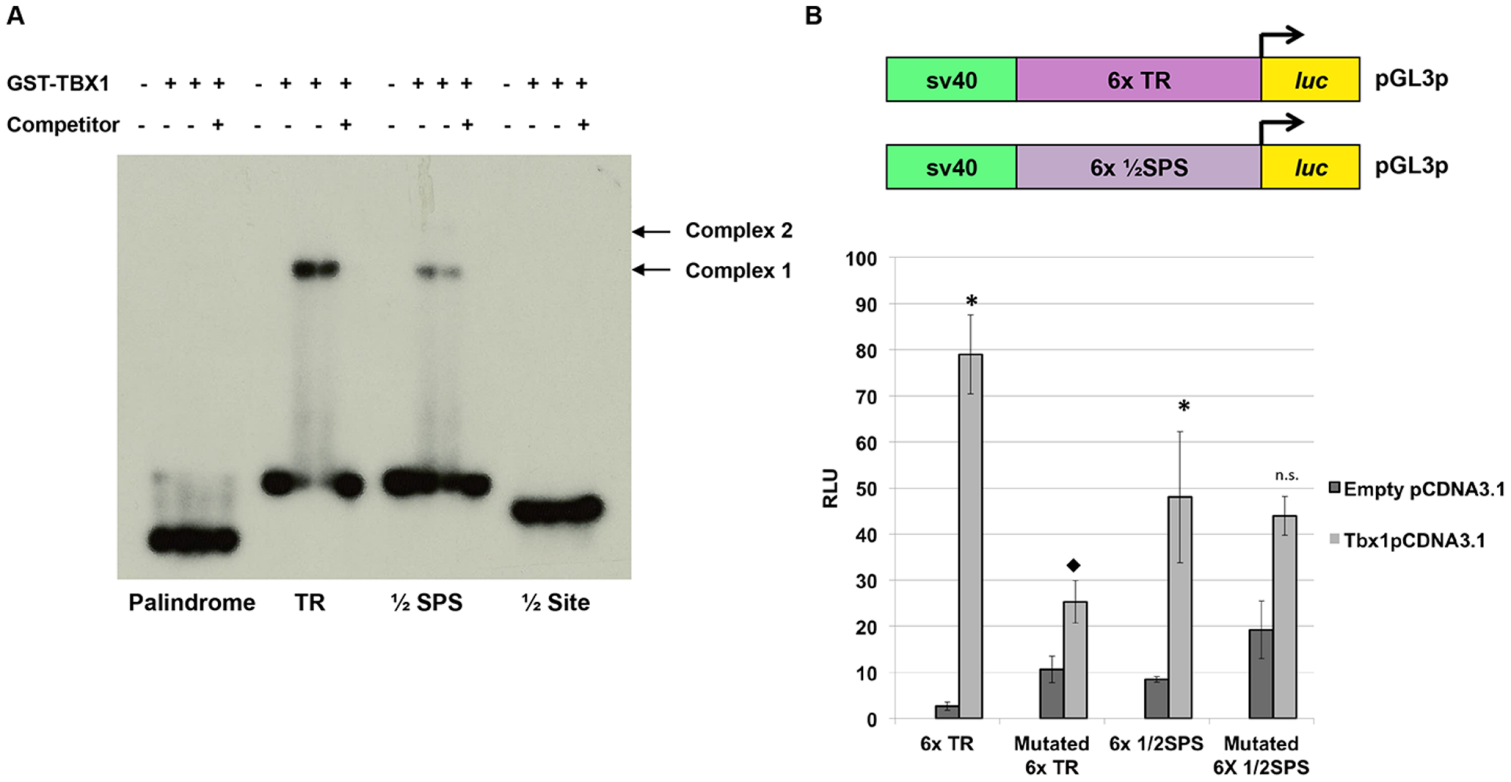
Specificity of new binding sites tested via EMSA and luciferase assays. A: The binding sites identified from the SELEX experiment were tested with GST-TBX1 separately by EMSA to determine if there is specific binding (Tandem Repeat, TR; Half Site Partial Site, ½SPS, half site; ½ Site). For comparison, the palindromic T-site was also tested but binding was very weak as compared to the newly identified binding sites and was only observed after extensive overexposure of the autoradiogram (not shown). B: Luciferase reporter constructs containing 6 copies (6x) of the TR and 6x of the ½SPS, respectively, were co-transfected with full length *Tbx1*-pCDNA3.1 and compared to the empty pCDNA3.1 transfection to determine if TBX1 could activate transcription of a reporter via these sites. A significant increase of luciferase activity was observed in the presence of full-length TBX1 for both the 6x TR and the 6x½SPS when compared to transfection of the empty pCDNA3.1 vector (TR: 29 fold; Students t test, *p<0.001; ½SPS: 5.6 fold; Students t-test, *p<0.02). The mutations analyzed were those previously tested in a half site where AGGTGTGA was mutated to AATTTTGA [31]. When these nucleotide changes were present in the TR, there was a dramatic decrease in activation by *Tbx1*-pCDNA3.1 (7.4 fold; Students t test, ♦p<0.001). The same mutation in the 6x½SPS construct did not show a significant change when compared to the normal ½SPS (n.s. not significant). All data are presented as means ±SD; n≥3.

To test for binding specificity, oligonucleotides were designed that contained a single copy of the TR and ½SPS, where each nucleotide corresponds to the most highly selected base at each position: TR: 5′-AGGTGTGAAGGTGTGA-3′ and ½SPS: 5′-AGGTGTGATCGCGTCAT-3′. The GST-TBX1 protein was able to bind to both motifs by EMSA (Fig. 2A). Two concentrations of protein were tested showing the same proportion of protein-DNA complexes (Fig. 2A). Binding was competed with 100X excess non-radiolabeled oligonucleotide of the same sequence. After exposing the film for an extended period of time, a second more slowly migrating protein-DNA complex appeared that was similar to that present in Fig.2A (data not shown). The shifted protein-DNA complex using the ½SPS appeared to be weaker in intensity on the gel as compared to the TR (Fig. 2A). In the same experiment, the 8bp half-site (T-site), 5′-AGGTGTGA-3′ and the *Brachyury* palindrome, 5-TCACACCTAGGTGTGAA-3′ were also tested. A protein-DNA complex was never observed when GST-TBX1 was incubated with the half-site and only after over exposing the film for 24 hours was a protein-DNA complex observed with the full Brachyury palindrome (data not shown). To further test the specificity of the newly derived motifs, gradients of both poly(dI-dC) and specific cold-competitor were generated and tested by EMSA (Fig. S2A). As the concentration of poly(dI-dC) increased, the binding intensity decreased; however, the protein-DNA complex was located at the same position in the gel, indicating specific binding occurred (Fig. S2A).

### TBX1 Transcriptionally Activates Reporter Genes

To test whether TBX1 could activate transcription of a reporter by binding to the newly identified consensus sequences, we performed luciferase assays using Jeg3 cells. Jeg3 cells have successfully been used previously to test TBX1 activation of reporter constructs harboring endogenous gene loci suggesting that it has the necessary co-factors for TBX1 to bind and regulate transcription [21]. Reporter constructs containing six tandem copies of the TR or ½SPS were generated and tested in luciferase assays (6xTR-pGL3p and 6x½SPS-pGL3p). After testing the full length TBX1 protein in pCDNA3.1, at varying concentrations, we found that 100 ng of the expression vector yielded the highest fold change (27 fold) when compared to empty pCDNA3.1 vector (data not shown). Based upon this, we used 100 ng of the *Tbx1-*pCDNA3.1 construct for all subsequent luciferase assays. The consensus sites and mutated versions of these sites (AGGTGTGA to AATTTTGA) [31], were simultaneously evaluated in the same experiment. As a control, for each binding site reporter construct, the *Tbx1-*pCDNA3.1 transfection was compared to the simultaneous transfection of empty pCDNA3.1 vector. The mutated 6xTR-pGL3p showed a dramatic decrease in transcriptional activation when compared to the wild-type (WT) reporter construct (Fig. 2B). The 6x½SPS-pGL3p also showed activation in the presence of *Tbx1-*pCDNA3.1 and this activation was only partially disrupted when the binding site was mutated (Fig. 2B).

### Surrounding Nucleotides Outside the Half Site are Crucial For Binding

**Figure 3:**
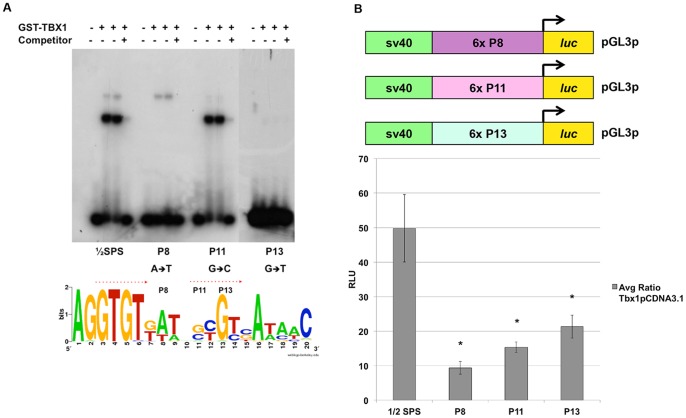
Surrounding nucleotides outside of the ½ Site are crucial for binding. A: EMSA was performed on mutated sequences that were generated in the second ½ site to test if variation at position, P8 (A→T), P11 (G→C) and P13 (G→T) affects binding. When P8 was changed, the faster migrating binding conformation was lost. When P11 was changed most of the slower migrating binding conformation was lost. Almost all binding was completely lost when P13 was changed. B: Luciferase reporter assays were performed to test their effect on transcription. Constructs harboring 6 copies of the mutated binding site (mutated at either P8, 11 or 13, respectively) were co-transfected with the full length *Tbx1* gene. Mutation of these nucleotides affected luciferase activation. Data are presented as means ± SD; n≥3. Student's t-test, *p<0.02.

To demonstrate the importance of the 3′ half of the TBX1-1/2 SPS site and to further define essential nucleotides for binding, we generated various mutations of position 8 (P8), 11 (P11) and 13 (P13) as these nucleotides seemed to vary the most when comparing complex intensities on gel shift assays (data not shown) (Fig. 3A). When the nucleotide at P8 was mutated from an A→T, binding of the lower, main protein-DNA complex (complex 1; Fig. 1A) was abolished. In contrast, the upper less prominent protein-DNA complex in the gel (complex 2; Figure 1A) remained unchanged (Fig. 3A). Mutation at P11 from a G→C nucleotide resulted in reduction of the upper protein-DNA complex (Fig. 3A). When the P13 nucleotide was mutated from a G→T, binding of GST-TBX1 was lost (Fig. 3A). Effects of these three mutations in the 2^nd^ half-site demonstrate the importance of these nucleotides in binding. We then proceeded to test these mutations in luciferase reporter assays in cell culture. We generated luciferase reporter constructs containing six copies of the mutated ½SPS at P8, P11 and P13. For all three, we observed reduced activation when compared to the WT ½SPS consensus sequence. We concluded that the surrounding nucleotides are necessary for the activation of the reporter in tissue culture (Fig. 3B; statistical values in Figure legend).

### Mutations in TBX1 Lead to a Decrease in Activation

**Figure 4:**
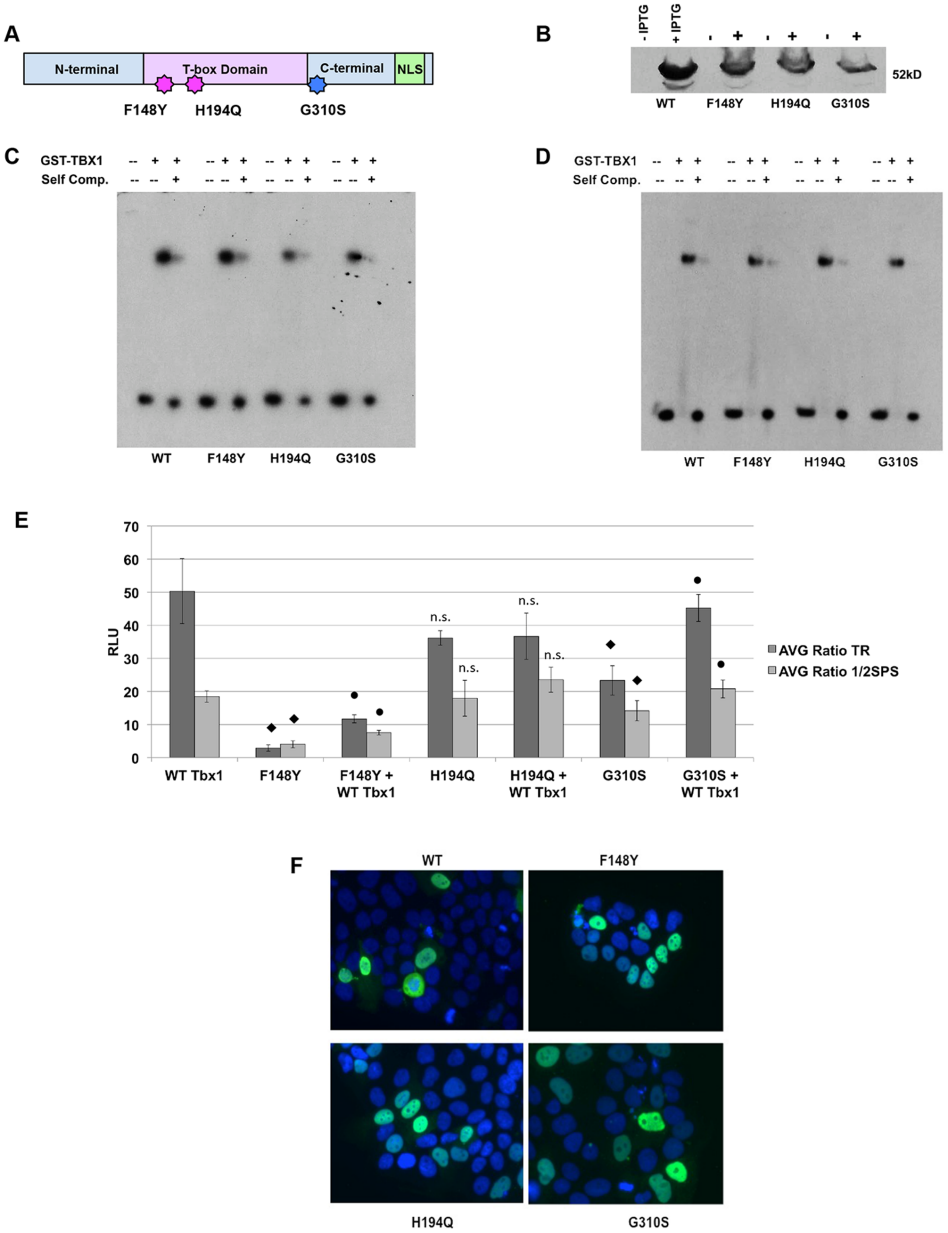
Human Mutations in Mouse Tbx1. A: The position of the three known mutations in *TBX1* in human patients are shown with respect to its domain structure [19]–[21]. The three mutations lie within the region that was cloned to generate the GST-TBX1 fusion protein. B: After mutagenesis, the wild type (WT) and mutant proteins were induced, purified and used for EMSAs. The protein-DNA complexes are shown before and after IPTG induction. C, D: EMSAs of the WT and three mutated proteins using the radiolabeled TR (C) and ½SPS motifs (D). E: Luciferase assays were performed after co-transfecting the reporter construct (6x TR or 6x ½ SPS) with WT or each mutated full length *Tbx1*-pCDNA3.1 construct. The F148Y mutation led to a decrease in activation (TR- 17 fold decrease, ♦p<0.0005; ½SPS- 4.5 fold decrease, ♦p<0.0003) when compared to the WT transfection. The H194Q mutation did not lead to a statistically significant change in activation but the trend was in the direction of decreased activity (not significant- n.s). The G310S mutation led to a smaller but still significant decrease in activation (TR- 2.2 fold decrease, ♦p<0.01; ½SPS- 1.3 fold decrease, ♦p<0.05). Equal amounts of WT and mutated *Tbx1* was co-transfected with the respective reporter constructs to determine if there was suppression of the mutant phenotype. There was a slight increase in activation when compared to the mutated F148Y alone transfection (TR-4 fold, •p<0.006; ½SPS- 1.9 fold, •p<0.006). Under these new conditions, the F148Y mutated TBX1 with WT protein still showed reduced activation when compared to WT TBX1 (TR- *p<0.0003; ½SPS- *p<0.006). The H194Q+WT combination did not show any significant change (TR- p<0.4; ½ SPS- p<0.1). The G310S+WT combination showed a significant increase in activation (TR- 2 fold, •p<0.001; ½SPS 1.5 fold, •p<0.02) when compared to G310S mutant alone. All data are presented as means ±SD; n≥3. p-values were determined using the Student's t-test. F: Immunoflourescence experiments were performed with antibodies to TBX1 on transfected Jeg3 cells to valdiate that the mutated constructs were localized to the nucleus (green). Nuclear localization was confirmed by observing expression in DAPI stained nuclei shown in blue.

Human mutations in *TBX1* have been previously identified in a subset of patients with VCFS/DGS but with no deletion. These mutations, including F148Y, H194Q and G310S, were previously tested in transcription reporter assays in cell culture using the Brachyury palindrome sequence to determine whether they altered transcription [19]-[21]. Increase in transcriptional activation with F148Y, H194Q and G310S mutations versus WT TBX1 using Jeg3 cells was previously observed [21]. To further test this using the new consensus sequences, we generated the same point mutations in GST-TBX1 and evaluated their DNA-binding and transcriptional activation in cell culture (Fig. 4A, 4B). Protein-DNA complexes were formed at the same position as for the WT protein (shift 1; Fig. 1A). Binding to DNA was similar with both consensus sequence probes as determined by EMSA (Fig. 4C and 4D). As for the wild-type protein, mutant TBX1 proteins could not bind to the half site (data not shown). In addition, luciferase reporter assays were carried out to determine if these mutations in TBX1 could lead to a change in the activation of the reporter constructs harboring the 6xTR or 6x½SPS. Cells were initially transfected with the test construct (WT or mutated TBX1) and the reporter construct. Mutated protein activation values were compared to the WT protein values. We observed a statistically significant decrease in activation in the presence of two mutations, F148Y and G310S; more dramatically with the F148Y mutation, which showed no activation when compared to WT values (statistical values are presented in the Figure legend). Interestingly, we did not observe any change in activation when we tested the H194Q mutation. Because these are heterozygous mutations in human patients, a reporter assay was carried out to test whether adding in one wild type copy of *Tbx1* would suppress the effect of the mutated allele. Jeg3 cells were co-transfected with 50 ng of *Tbx1-*pCDNA3.1 and 50 ng of either F148Y, H194Q or G310S *Tbx1-*pCDNA3.1 as we described earlier. Although there was a slight increase in activation in the presence of the WT protein with the G310S mutant protein, these values were still lower for the F148Y mutations when compared to the WT protein. Again, there was no change observed when the WT TBX1 protein was co-transfected with the H194Q mutant protein. (Fig. 4E). Because these recombinant proteins are not endogenously expressed in Jeg3 cells, we examined whether the ectopic TBX1 proteins were localized to the nucleus and not in the cytoplasm. Immunofluorescence was performed to visualize this set of four proteins and their nuclear localization was confirmed, using DAPI as a nuclear stain (Fig. 4F). We concluded that transfection conditions that mimic TBX1 haploinsufficiency due to the F148Y mutation resulted in reduced activation of both reporter constructs.

### In Silico Genome-Wide Screen for T-sites in the Mouse Genome

**Figure 5:**
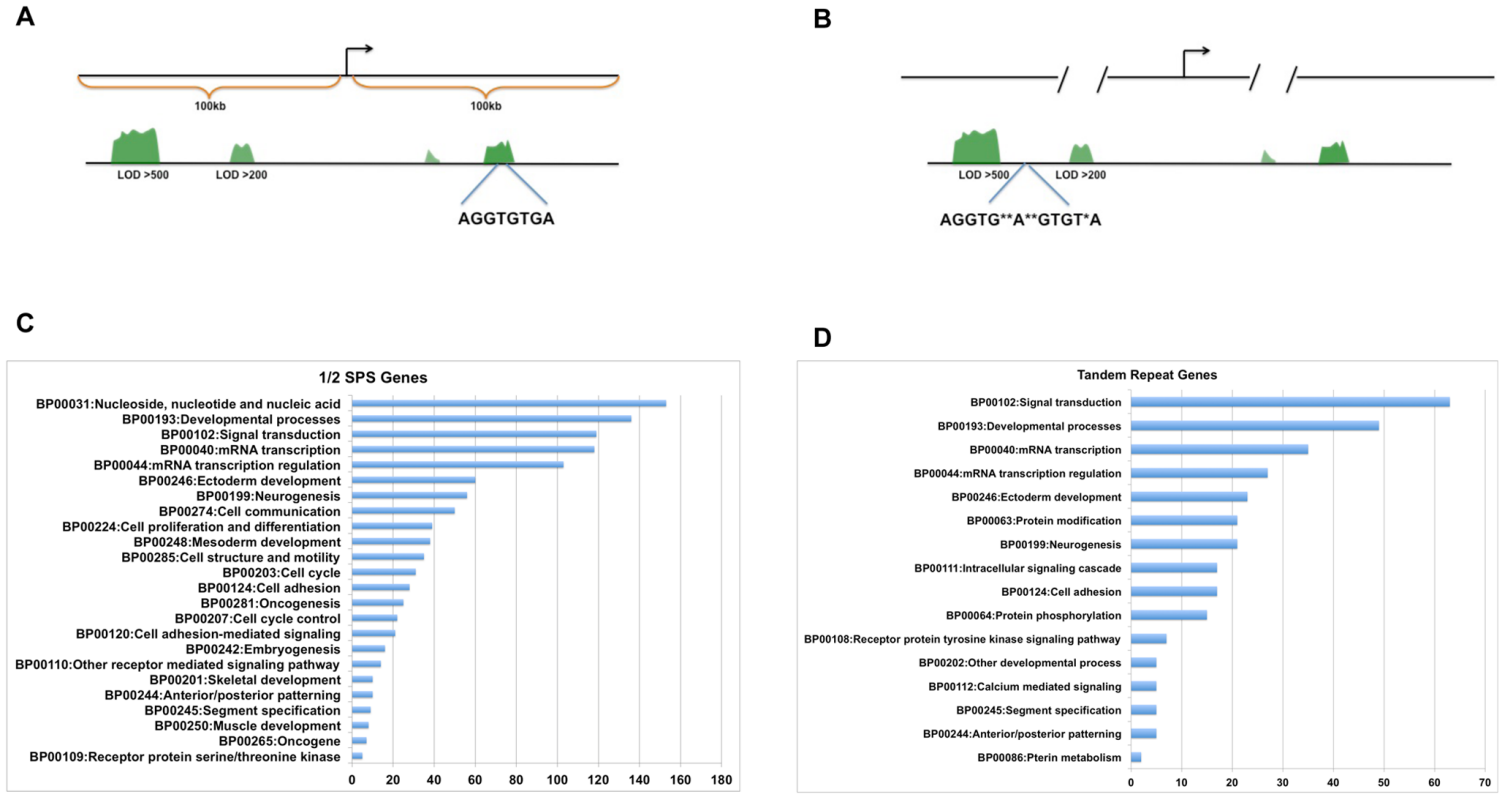
Genome wide search of T-sites. A, B: Representative examples depicting the genome-wide search for ½ SPS and TR motifs. The first search was done for the ½ site in evolutionarily conserved blocks within 100 kb upstream and downstream of the TSS of genes. The second search for the TR was expanded since there were fewer sites (303), irrespective of distance to the TSS and conservation across mammals or vertebrates. C, D: Bar graphs depict the number of genes comprising top gene ontology categories using the Database for Annotation, Visualization and Integrated Discovery (DAVID v6.7- http://david.abcc.ncifcrf.gov).

A series of bioinformatic approaches were undertaken to identify potential direct downstream transcriptional target genes by examining annotated mouse genome sequence data (UCSC genome browser, mm9). The first screen was done to detect binding sites in blocks of evolutionarily conserved sequences. A total of 235,414 sequences matching half sites were found. Among them, 12,659 (5.4%) half sites were found to overlap with conserved elements (Fig. 5A) These were then assigned to the nearest RefSeq genes within +/−100 kb distance of transcriptional start sites (TSS). The first screen identified a total of 187 with matches to the half site consensus sequence of AGGTG(T/C)(G/T)A within highly conserved elements (Logarithm of the Odds Score-LOD>500) (Motifs and corresponding gene names can be found in Table S1). We found 425 motifs within moderately conserved elements (LOD score of 200–500) (Table S1). These could be putative binding sites for any T-box gene. We then searched the half sites that contained the second partial site, to identify ½SPSs, which would be more selective for binding of TBX1. We also examined whether any of the half sites had a second direct tandem repeat. None of the sites within evolutionarily conserved blocks from the search above had a second direct repeat (TR). Therefore, a second bioinformatic screen was done to search for TR sites anywhere in the genome, irrespective of evolutionary conservation (Fig. 5B). A total of 302 TR sites were found throughought the genome (Table S2). We searched for gene ontology groups for all of the genes harboring putative T-half sites and TR sites. Most of the groups that were identified were those involved in embryonic develpmental processes and mRNA transcription regulation (Fig. 5C,D) [46], [47]. We then examined each gene for their known function or expression pattern, using literature and the MGI JAX database (www.informatics.jax.org) to ascertain whether any could be a putative TBX1 downstream transcriptional target (data not shown).

Candidate TBX1 binding sites near genes with known expression patterns in mouse embryos similar to that of *Tbx1* or with phenotypes similar to that in *Tbx1^-/-^* mutant embryos were of particular interest to pursue. To further narrow the list of possible genes regulated by *Tbx1*, we focused on those containing ½SPS or TR sites. We first checked whether the expression of any were altered in previous gene expression arrays in experiments where *Tbx1^+/+^* versus *Tbx1^-/-^* embryonic tissues were compared [26], [28]. Twenty-seven genes were initially selected to determine if GST-TBX1 could bind.

Electrophoretic mobility shift assays were performed on motifs near candidate downstream target gene loci to determine if GST-TBX1 could bind to them. GST-TBX1 formed protein-DNA complexes with 19 of the 27 motifs with three distinct intensities of protein-DNA complexes referred to as high (similar to TBX1-TR), medium and low (Table S3). The position of the complexes in the gel were all the same, suggesting similar binding conformations. Reporter constructs were generated to include the motif to be tested with approximately 200 bp flanking either side. Of the 19 motifs tested for binding, we chose three of the strongest binding candidates for additional studies: *Fgf8*, *Bmper* and the *Otog-MyoD* locus.

**Figure 6:**
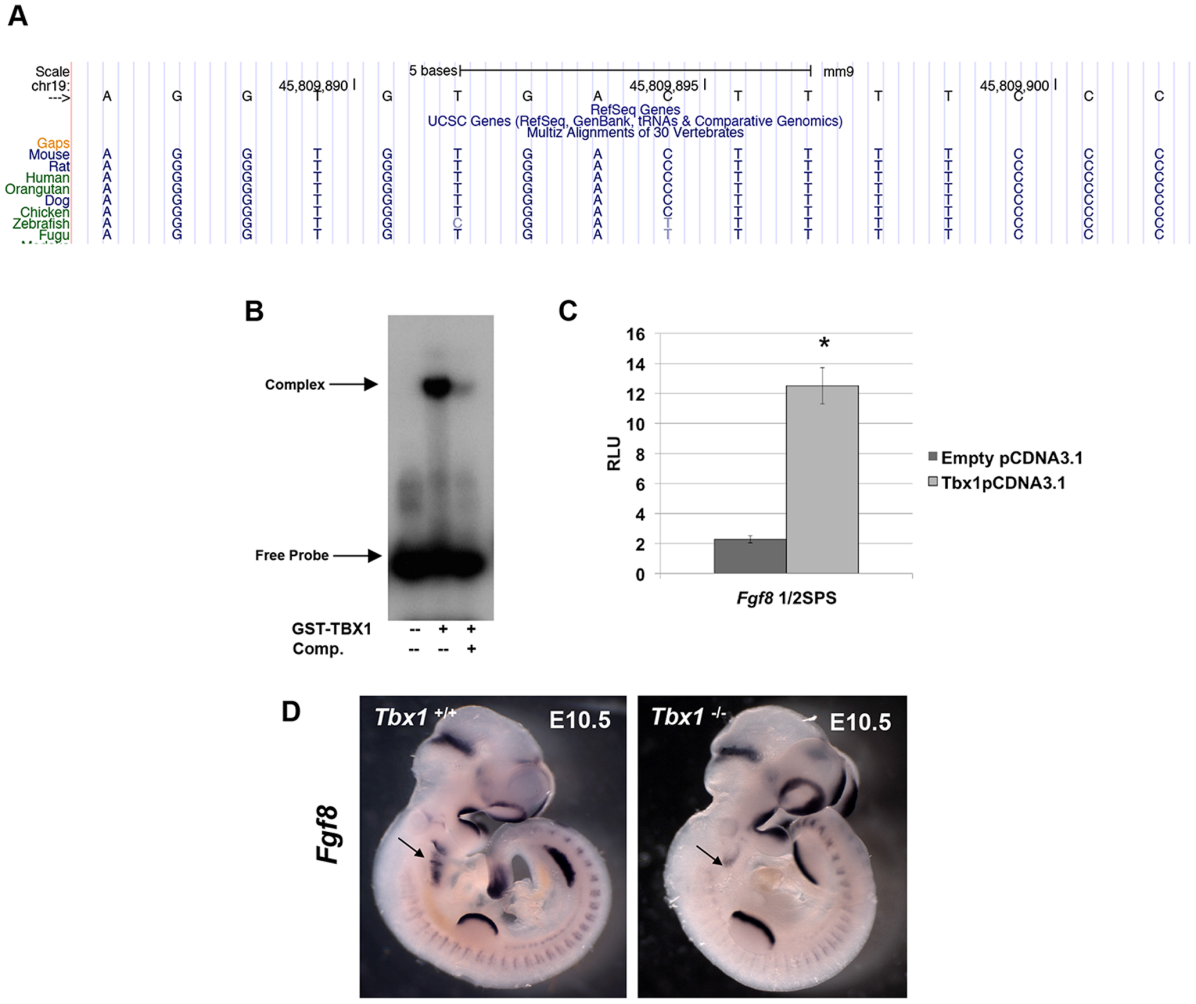
The *Fgf8* locus has a ½SPS that is bound and activated by TBX1. A: Snapshot from the UCSC Genome Browser showing the position of the ½SPS, located 4 kb downstream of the *Fgf8* gene. The region is ultraconserved from humans to fugu. B: EMSA for the 40 bp element in the *Fgf8* locus harboring the ½SPS motif with GST-TBX1. Lanes with unlabeled competitor is shown. C: The 400 bp element within *Fgf8* locus was subjected to luciferase assays in cell culture and was activated in the presence of *Tbx1*-pCDNA3.1 (5-fold; Students t- test *p<0.002; data are presented as means ±SD; n≥3). D: Whole mount *in situ* hybridization of *Fgf8* antisense mRNA in *Tbx1^+/+^* (left) versus *Tbx1* ^-/-^ (right) mouse embryos at E10.5. Expression of *Tbx1* is reduced in the distal pharyngeal apparatus (arrow), but remains in the rest of the embryo (first pharyngeal arch, head, limb buds, somites).

The *Fgf8* gene encodes a secreted fibroblast growth factor (FGF) that is required for craniofacial and heart development [45]. Relevant to *Tbx1*, a genetic interaction between *Fgf8* and *Tbx1* has been found [29], [45]. The *Fgf8* locus has a ½ SPS located 4 kb downstream of the transcriptional stop site (Fig. 6A). This site falls in a highly evolutionarily conserved sequence block, across mammals and vertebrates. Interestingly this is a known *Fgf8* regulatory region for somite and tail bud mRNA expression, conserved from zebrafish to mouse [48], [49]. GST-TBX1 was able to bind to the ½SPS motif (Fig. 6B) and this was at a similar intensity as compared to the consensus ½SPS (AGGTGTGATCGCGTCAT) (data not shown). As expected from the EMSA, the transcription reporter assay in Jeg3 cells showed activation at a level similar to the ½ SPS consensus (5 fold change) (Fig. 6C). Whole mount RNA *in situ* hybridization comparing *Tbx1^+/+^* and *Tbx1* ^-/-^ embryos at E10.5 shows a decrease in expression of *Fgf8* in the pharyngeal arch endoderm (Fig. 6D) as previously reported [29], [50]. We did not detect a change in somite or tail bud expression, suggesting possible functional redundancy with other T-box genes with similar expression patterns.

**Figure 7:**
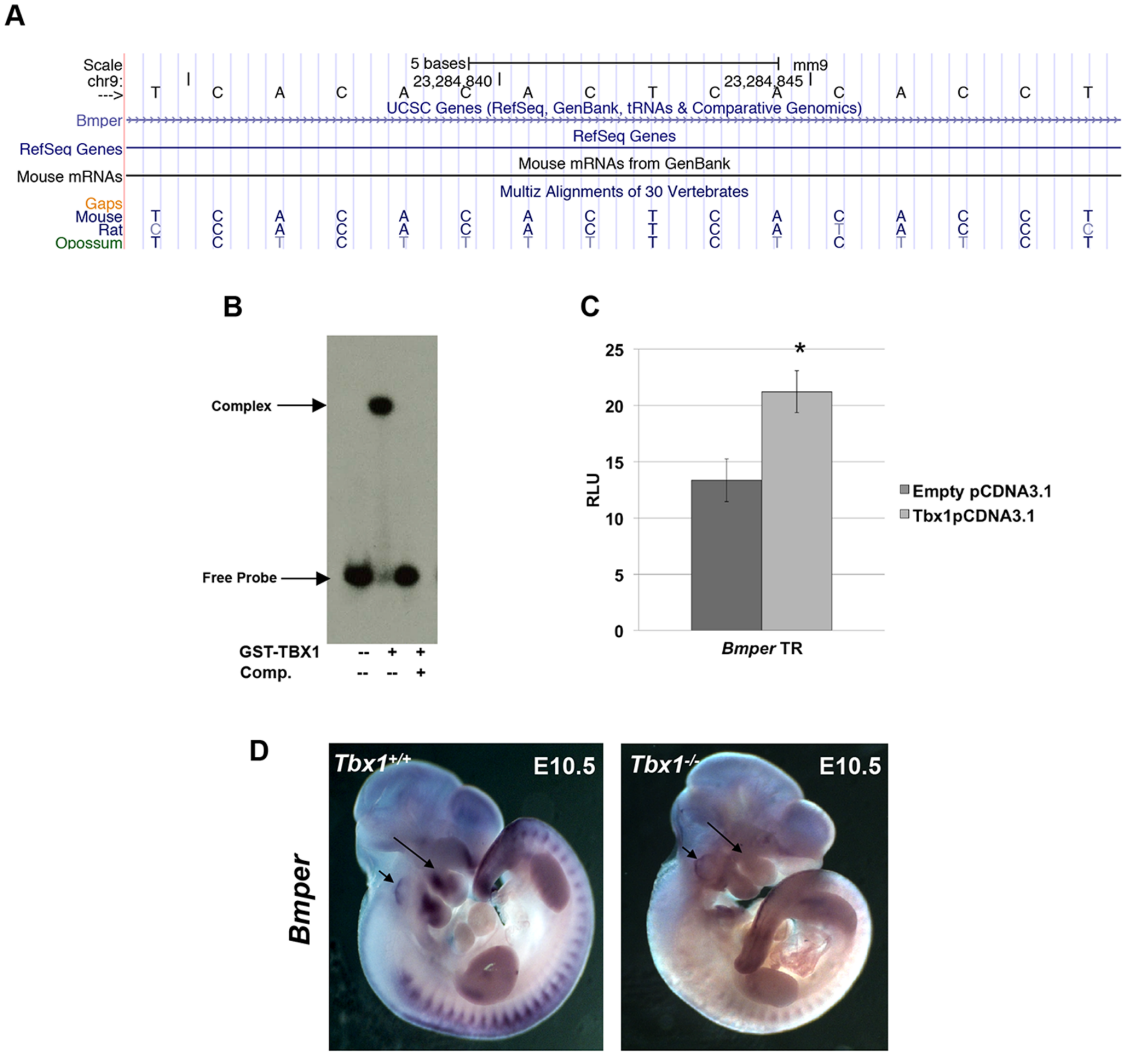
The *Bmper* locus has an intronic TR site that is bound and activated by TBX1. A: Snapshot of the UCSC browser showing the TR site in intron 13 in the *Bmper* locus and it is evolutionarily conserved in some rodent species. B: GST-TBX1 was able to bind to the TR motif in the *Bmper* locus. C: Luciferase assay results demonstrate a 1.8 fold increase (Students t-test *p<0.003; ±SD; n≥3) in activation when *Tbx1*pCDNA3.1 was cotransfected with the 400 bp element. D: Whole mount *in situ* hybridization of *Bmper* antisense mRNA in *Tbx1^+/+^* (left) versus *Tbx1^-/-^* (right) embryos at E10.5. Expression of *Tbx1* is reduced in the core mesoderm of the pharyngeal apparatus (long arrow) and otic vesicle (short arrow), but remains in the somites.

The *Bmper* gene encodes a secreted protein that inhibits bone morphogenetic protein (BMP) function. The *Bmper* locus has a TR binding site in the intron lying between exons 13 and 14, sharing evolutionary conservation only with rat and opossum (Fig. 7A). GST-TBX1 was able to bind strongly to the 40 bp element harboring the motif (Fig. 7B) and this was similar to that of the TR consensus (data not shown). There was a small 1.8 fold increase in transcription activation in the presence of TBX1 (Fig. 7C). We suggest that the small fold activation here compared to the experiments using the 6x TR consensus sequence, could be due to the fact that we used a 400 bp sequence element that might harbor inhibitory sites surrounding the single TBX1 binding site. *Bmper* expression is lost in part of the first pharyngeal arch in *Tbx1 ^-/-^* embryos at E10.5, and expression in the inner ear is altered as well (Fig. 7D) suggesting that it could be a direct downstream transcriptional target.

**Figure 8:**
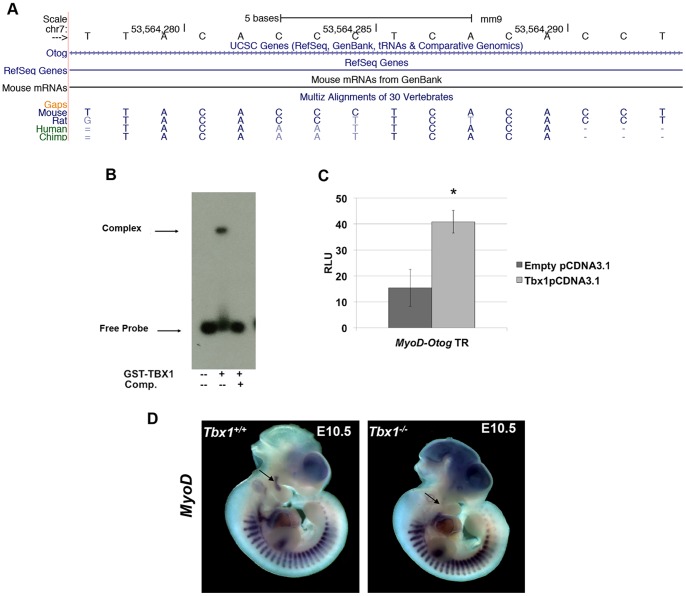
TBX1 can bind to a TR site in the *Otog-MyoD* locus. A: Snapshot from the UCSC genome browser showing the TR site in intron 13 of the *Otog* gene and 77 kb downstream from the TSS of *MyoD*. B: EMSA of GST-TBX1 and the TR motif in the *Otog-MyoD* locus with or without unlabeled TR double stranded oligonucleotide competitor. C: Luciferase reporter assays using the intronic element showed a 2.5 fold increase (Student's t-test, *p<0.05; means ±SD; n≥3) in activation when in the presence of TBX1. D: Expression of *MyoD* is lost in the 1^st^ pharyngeal arch core mesoderm in *Tbx1* ^-/-^ embryos at E10.5 (arrow) but it remains in the somites. Forebrain expression in both embryos represents a staining artifact.

The third site that was evaluated was a TR site in the *Otog* gene in intron 53 (of a total of 56 exons). The *Otog* gene encodes an N-glycosylated protein present in the acellular membranes of the sensory epithelia patches of the inner ear, important for hearing [51]. *Otog* and *MyoD* are neighboring genes, however the TR motif within the regions tested in EMSA and luciferase assays, is 70 kb from the *MyoD* TSS (Fig. 8A). The GST-TBX1 fusion protein can bind to the *Otog-MyoD* sequence block containing the TR motif, and it was competed with unlabeled DNA of the same sequence. Although binding appeared to be strong by EMSA, transcription was only activated 2.5 fold (Fig. 8B and 8C). The *MyoD* gene, encoding a basic helix-loop-helix myogenic regulatory transcription factor lies adjacent to *Otog*. We were not able to generate a specific probe for *in situ* hybridization analysis of *Otog*. As has been reported, RNA expression of *MyoD* is lost in the first pharyngeal arch core mesoderm in *Tbx1^-/-^* null mutant embryos [52] (Fig. 8D). We conclude that this endogenous TR site is a possible candidate for *MyoD* regulation by TBX1.

## Discussion

The T-box family of transcription factors is important in vertebrate development and human disease. The preferential binding site of a number of T-box proteins, including Brachyury, TBX2, TBX5, TBX6, TBX15 and TBX18 were previously identified by taking either *in vitro* or *in vivo* approaches [4],[12],[14]–[16]. Most can bind as monomers to a Brachyury consensus half site, or as dimers to a palindrome, while few can also bind to a tandem repeat. In this study we carried out an *in vitro* selection method (SELEX) to identify the preferential binding site of mammalian TBX1. We found two classes of binding sites; a perfect direct repeat (TR), consisting of two classic Brachyury half-sites and a second, imperfect direct repeat (½SPS), in which the 5′ site is similar to the Brachyury half site, but the 3′ half is different. One important possibility is that there are differences in the amino acid constitution of TBX1 that confers a different binding preference as some of the other T-box proteins. For example, TBX1 appears to bind strongly to the TR, but weakly to the Brachyury palindrome and not at all to the half site motif. Although the DNA binding domain is highly conserved amongst different T-box proteins, some differences may contribute to specificity of binding and the sequences might affect the orientation in which various T-box proteins bind to DNA. Interestingly, a few amino acids that are important for Brachyury dimers to bind to the palindrome are not conserved in TBX1 [20], [53]. This may explain the difference in binding preference. Using the Brachyury crystal structure, amino acids important for both the dimerization and DNA binding have been mapped [53]. Six amino acids important for binding and dimerization, distributed throughout the protein, are different between Brachyury and TBX1. Three of these amino acids are important for dimerization (M87D, N131A, F132K) and three important for DNA binding (K103R, K151N, A216G; Brachyury to TBX1 cid change respectively). Perhaps, these differences at crucial positions leads to a secondary structure conformational change allowing TBX1 molecules to bind preferably in a head to tail orientation. The TBX1 protein and DNA crystal structure has been published, but this was done so using the palindromic Brachyury binding site [54]. This group found that two TBX1 proteins can bind as monomers to the palindromic sequence. In our gel shift assays, TBX1 and Brachyury formed similar sized protein-DNA complexes (data not shown), suggesting that TBX1 might bind as a dimer to the TBX1 TR, since it consists of two half-sites. Now that the TBX1 TR has been identified as the preferential binding site, a new crystal structure might lead to further understanding of key residues of TBX1 required for binding to DNA.

We found some inconsistencies between apparent binding affinities to DNA in EMSAs versus transcriptional activity in luciferase assays in cell culture. For example, although the P13 mutation in the ½SPS led to the greatest loss of binding *in vitro*, it had the least effect on transcription (2.3 fold decrease). One possible reason is that we used only the DNA binding domain of TBX1 for EMSAs but used the full-length protein for luciferase assays. There are multiple examples where there is a lack of direct correlation between relative binding affinity and transcriptional activation of a cis-acting motif [55]. For example, the ETS-1 DNA binding domain (DBD) undergoes minimal secondary structural changes in the presence of DNA, but the full length protein binding to DNA does induce changes in secondary structure at a distance from the protein-DNA interference [56]. Glucocorticoid receptor binding also affects structure and activity of the protein on DNA where stronger activating downstream sites bound equally in gel shift assays as those more weakly activated in luciferase assays [57]. It was also noted that changing even one nucleotide in the binding sequence could affect the binding and transcriptional activation. DBDs are not only important for protein-DNA interactions, but for protein-protein interactions as well. Perhaps GST-TBX1, in the presence the P13 binding site, has a more open interface allowing it to interact with other co-factors that provide for a more stable activation of transcription as opposed to the P8 or P11 nucleotide changes.

### Mutations in TBX1

Previous studies have tested whether *TBX1* mutations have an impact on transcriptional regulation of reporter constructs, but these used palindromic sequences as the binding motif, but these had conflicting results [20], [21]. Since the binding consensus sites identified in the SELEX assay had a roughly, 20 fold increase in binding and transcription activation by TBX1, we reasoned that it would provide a more sensitive indicator of any change in binding or transcription by missense changes in *TBX1*. Previous studies did not perform any *in vitro* binding assays to DNA. We found that the F148Y, H194Q and G310S mutant proteins could strongly bind to the two consensus sequences we identified (TR and ½SPS sites). In contrast to what has been previously reported, where the F148Y and G310S mutations showed no effect on transcription [19] or the three (F148Y, H194Q, G310S) showed an increase in transcription of reporters using the palindrome site [21], we found a decrease in reporter gene activation, in particular for the F148Y mutation. This suggests that activation or repression might be DNA binding motif-specific. One possible explanation of this difference with previous reports [19]–[21] is that the new consensus sequence(s) provides a greater sensitivity in measuring changes in the mutant proteins. Based on the published TBX1 crystal structure, F148 and H194 are neither involved with DNA binding or dimer formation, but it was noted that F148 lies at the surface of the protein [54]. The authors explain that this residue then may have an effect on protein-protein interactions with other co-factors necessary for transcription to occur [54]. This coincides with our data in which the F148Y mutation does not affect binding to DNA (Figs. 4C, D), but does lead to a loss of activation in reporter assays (Fig. 4E).

In addition to understanding the effects of mutations on gene function, one major goal is to identify direct transcriptional target genes required for embryonic development. Using various bioinformatic selection methods, we identified and validated DNA binding to 19 different motifs present in gene loci of interest, and confirmed transcriptional activation for 11 of them (data not shown), including *Fgf8*, *Bmper* and *Otog-MyoD*.

### Fgf8

The pharyngeal apparatus is an embryonic structure that becomes remodeled to form the face, neck and cardiac outflow tract [58]. *Tbx1* is expressed in mouse embryos in the endoderm and mesoderm of the pharyngeal arches as well as the ectoderm of the distal pharyngeal apparatus, with some localized expression in the somites [26], [45]. *Tbx1* and *Fgf8* are coexpressed in the pharyngeal endoderm and a subset of the pharyngeal mesoderm and they genetically interact in mouse embryos, implicating them in the same genetic pathway [29] [50]. An evolutionarily conserved element has been identified downstream of the *Fgf8* gene locus and drives expression of a reporter in forebrain, somites and tail bud but not the pharyngeal apparatus [48]. *Tbx1* and *Fgf8* are also expressed in the somites and presomitic mesoderm (PSM). However, inactivation of *Tbx1* does not result in loss of *Fgf8* expression in these tissues nor does it affect development of these structures. The most parsimonious explanation is that *Tbx1* acts redundantly with other T-box genes, upstream of *Fgf8*. There are several T-box genes expressed in the somites and tail bud [59]–[61] and one of these may in fact regulate transcription, possibly Brachyury, which can also bind to direct repeats or TBX6 whose preferential binding site has some resemblence to the ½SPS [15].

### *Bmper, Otog-MyoD* and transcription regulation

Although many known regulatory regions show evolutionary conservation, not all follow this pattern. We found putative TBX1 protein binding sites in the *Bmper* and *Otog-MyoD* gene loci, however, they are not in regions of high evolutionary conservation. There have been a number of reports examining regulatory regions that are not in conserved elements. For example, many of the p300 sites found by ChIP-Seq (chromatin immunoprecipitation followed by next-generation sequencing) were not in evolutionarily conserved blocks, however, they did drive expression of reporters *in vivo* [62]. The same was true for PHOX2B direct downstream target genes found in zebrafish [63]. Recent investigation of specific binding by liver transcription factors in five vertebrate species have shown that occupancy of a small minority (10%–22%) of the binding sites on DNA is conserved among mammalian species [64]. Non-conserved, biologically functional enhancers have also been identified upstream of *pax9* and *otx1b* in zebrafish [65]. Changes in transcriptional programs through the changes within non-conserved regions are supposed to drive evolution [66]. Therefore, we suggest that the TR sites warrant careful investigation. To this regard, we analyzed such sites in the *Bmper* and the *Otog-MyoD* loci.

The core mesoderm of the pharyngeal arches form the muscles of the craniofacial region and neck, required for chewing and swallowing [58]. We found that the *Bmper* gene, encoding a BMP antagonist [67]–[69] is strongly expressed in the central core mesoderm region of the pharyngeal arches. The first pharyngeal arch forms, but the distal arches do not form in *Tbx1* ^-/-^ embryos. We found that Bmper mRNA expression is lost in the first pharyngeal arch in *Tbx1* ^-/-^ embryos.

Similar to *Bmper*, another gene, *MyoD* is also expressed in the core mesoderm of the pharyngeal arches and it is also reduced in expression in *Tbx1* ^-/-^ embryos. Inactivation of *MyoD* and other basic helix-loop-helix regulatory transcription factors, results in loss of craniofacial muscle formation [70], [71]. Similarly, inactivation of *Tbx1* results in loss of development of craniofacial muscles [52], [72]. The motif we identified is within the *Otog* gene body, but near the 3′ end of the gene. *Otogelin (Otog)* encodes a glycoprotein present in the acellular gelatinouses structures covering the sensory epithelia of the inner ear [51], [73]. It is known that *Otog* is expressed in the inner ear as early as E10. Mutations in *Otog* lead to autosomal-recessive sensorineural nonsyndromic hearing loss, showcasing the importance of this protein in inner ear development and hearing [74]. Unfortunately, we were not able to generate an RNA anti-sense probe to *Otog* to determine if it is co-localized with*Tbx1*. On the other hand, it is possible that this site is important for regulation of *MyoD* expression *in vivo*, that these sequences are important for regulation of *Otog*, or neither. Nearby enhancers within neighboring genes have been found to regulate genes at a distance. This is the case for *Dlx5*, with two enhancers being exons of the neighboring gene *Dync1/1* [75]. Only future ChIP and *in vivo* reporter assays in transient transgenic mouse models can validate this hypothesis. In conclusion, we used an *in vitro* SELEX selection process to identify two novel TBX1 consensus sequences, the TBX1-TR and the TBX1-½SPS. We found that TBX1 can activate reporter constructs harboring the newly identified binding sites in tissue culture. In addition, we have also demonstrated that in the presence of the F148Y human mutation in *TBX1*, activation of reporter constructs was strongly diminished. This was only possible having a highly active consensus site for transcription reporter assays in cell culture. Finally, as a prelude to future ChIP-seq and other biochemical studies, we provide an *in silico* list of possible direct downstream target genes, some of which may be biologically relevant to TBX1 function, such as *Fgf8*, *Bmper* and *Otog-MyoD*.

## Supporting Information

Figure S1 **Cloning of GST-Tbx1 (T-box) Construct.** A: The T-box region (amino acids 90–303) of mouse *Tbx1* was PCR amplified from cDNA with flanking EcoRI and XhoI restriction enzyme sties. These sites were used to subclone the fragment into the pGEX4t3 vector (GE Healthcare) to generate a GST-TBX1 fusion protein. B: GST-TBX1 was detected via western blot with an α GST antibody, with an approximate molecular weight of 52 kD. C: EMSA with recombinant GST-TBX1 (90–303) binds to published palindromic Brachyury palindrome motif [12]. Protein dilution, 1∶1 and exposure time, 6 hrs. Probe: CTAGATTTCACACCTAGGTGTGAAATCTAG.
(TIF)

Figure S2 **Testing the binding specificity of TBX1 binding motifs.** Gradients of both poly dI-dC (left) and specific cold-competitor (right). As the concentration of the poly dI-dC increased (0.5–2 µg), the binding decreased in intensity but the creation of protein-DNA complexes still occurred at the same position. Increasing amounts of specific cold-competitor (25x-200x) was used to demonstrate the specificity of binding.
(TIF)

Table S1 **Sites and nearest genes with T half site.** The sites listed below are half-sites that lie in evolutionarily conserved regions across the mouse genome (mm9).
(XLS)

Table S2 **Sites and nearest genes with tandem repeat site.** The sites listed below are TR sites across the mouse genome (mm9), irrespective of evolutionary conservation.
(XLSX)

Table S3 **TR and ½ SPS endogenous sites in the mouse genome.** The sites listed above are the endogenous sites that were bound by GST-TBX1 in EMSA experiments. Listed as well is the distance to the TSS and comparative gel shift band intensity. *Gel shift bands were compared to the binding of TBX1 to the TR TBX1 site (High).
(TIF)

## References

[1] Dobrovolskaia-Zavadskaia N (1927) Sur la motificationsponta-nee de la queue chez la souris nouveau-nee et sur l'existence d'un caractere hereditaire ‘non viable’. cr hebd Soc Biol 97: 3.

[2] Chesley P (1935) Development of the short-tailed mutant in the house mouse. The Journal of Experimental Zoology 70: 7.

[3] Gluecksohn-Schoenheimer S (1938) The Development of Two Tailless Mutants in the House Mouse. Genetics 23: 573–584. 17246902 10.1093/genetics/23.6.573 PMC1224290

[4] Kispert A, Herrmann BG (1993) The Brachyury gene encodes a novel DNA binding protein. EMBO J 12: 3211–3220. 8344258 10.1002/j.1460-2075.1993.tb05990.x PMC413588

[5] Chapman DL, Garvey N, Hancock S, Alexiou M, Agulnik SI, et al (1996) Expression of the T-box family genes, Tbx1-Tbx5, during early mouse development. Dev Dyn 206: 379–390. 8853987 10.1002/(SICI)1097-0177(199608)206:4<379::AID-AJA4>3.0.CO;2-F

[6] Bollag RJ, Siegfried Z, Cebra-Thomas JA, Garvey N, Davison EM, et al (1994) An ancient family of embryonically expressed mouse genes sharing a conserved protein motif with the T locus. Nat Genet 7: 383–389. 7920656 10.1038/ng0794-383

[7] Agulnik SI, Garvey N, Hancock S, Ruvinsky I, Chapman DL, et al (1996) Evolution of mouse T-box genes by tandem duplication and cluster dispersion. Genetics 144: 249–254. 8878690 10.1093/genetics/144.1.249 PMC1207498

[8] Naiche LA, Harrelson Z, Kelly RG, Papaioannou VE (2005) T-box genes in vertebrate development. Annu Rev Genet 39: 219–239. 16285859 10.1146/annurev.genet.39.073003.105925

[9] Bamshad M, Lin RC, Law DJ, Watkins WC, Krakowiak PA, et al (1997) Mutations in human TBX3 alter limb, apocrine and genital development in ulnar-mammary syndrome. Nat Genet 16: 311–315. 9207801 10.1038/ng0797-311

[10] He M, Wen L, Campbell CE, Wu JY, Rao Y (1999) Transcription repression by Xenopus ET and its human ortholog TBX3, a gene involved in ulnar-mammary syndrome. Proc Natl Acad Sci U S A 96: 10212–10217. 10468588 10.1073/pnas.96.18.10212 PMC17868

[11] Packham EA, Brook JD (2003) T-box genes in human disorders. Hum Mol Genet 12 Spec No 1: R37–44. 10.1093/hmg/ddg077 12668595

[12] Sinha S, Abraham S, Gronostajski RM, Campbell CE (2000) Differential DNA binding and transcription modulation by three T-box proteins, T, TBX1 and TBX2. Gene 258: 15–29. 11111039 10.1016/s0378-1119(00)00417-0

[13] Wilson V, Conlon FL (2002) The T-box family. Genome Biol 3: REVIEWS3008. 12093383 10.1186/gb-2002-3-6-reviews3008 PMC139375

[14] Ghosh TK, Packham EA, Bonser AJ, Robinson TE, Cross SJ, et al (2001) Characterization of the TBX5 binding site and analysis of mutations that cause Holt-Oram syndrome. Hum Mol Genet 10: 1983–1994. 11555635 10.1093/hmg/10.18.1983

[15] White PH, Chapman DL (2005) Dll1 is a downstream target of Tbx6 in the paraxial mesoderm. Genesis 42: 193–202. 15986483 10.1002/gene.20140

[16] Farin HF, Bussen M, Schmidt MK, Singh MK, Schuster-Gossler K, et al (2007) Transcriptional repression by the T-box proteins Tbx18 and Tbx15 depends on Groucho corepressors. J Biol Chem 282: 25748–25759. 17584735 10.1074/jbc.M703724200

[17] Conlon FL, Fairclough L, Price BM, Casey ES, Smith JC (2001) Determinants of T box protein specificity. Development 128: 3749–3758. 11585801 10.1242/dev.128.19.3749

[18] Kispert A, Koschorz B, Herrmann BG (1995) The T protein encoded by Brachyury is a tissue-specific transcription factor. EMBO J 14: 4763–4772. 7588606 10.1002/j.1460-2075.1995.tb00158.x PMC394574

[19] Stoller JZ, Epstein JA (2005) Identification of a novel nuclear localization signal in Tbx1 that is deleted in DiGeorge syndrome patients harboring the 1223delC mutation. Hum Mol Genet 14: 885–892. 15703190 10.1093/hmg/ddi081

[20] Yagi H, Furutani Y, Hamada H, Sasaki T, Asakawa S, et al (2003) Role of TBX1 in human del22q11.2 syndrome. Lancet 362: 1366–1373. 14585638 10.1016/s0140-6736(03)14632-6

[21] Zweier C, Sticht H, Aydin-Yaylagul I, Campbell CE, Rauch A (2007) Human TBX1 missense mutations cause gain of function resulting in the same phenotype as 22q11.2 deletions. Am J Hum Genet 80: 510–517. 17273972 10.1086/511993 PMC1821102

[22] Merscher S, Funke B, Epstein JA, Heyer J, Puech A, et al (2001) TBX1 is responsible for cardiovascular defects in velo-cardio-facial/DiGeorge syndrome. Cell 104: 619–629. 11239417 10.1016/s0092-8674(01)00247-1

[23] Jerome LA, Papaioannou VE (2001) DiGeorge syndrome phenotype in mice mutant for the T-box gene, Tbx1. Nat Genet 27: 286–291. 11242110 10.1038/85845

[24] Lindsay EA, Vitelli F, Su H, Morishima M, Huynh T, et al (2001) Tbx1 haploinsufficieny in the DiGeorge syndrome region causes aortic arch defects in mice. Nature 410: 97–101. 11242049 10.1038/35065105

[25] Ivins S, Lammerts van Beuren K, Roberts C, James C, Lindsay E, et al (2005) Microarray analysis detects differentially expressed genes in the pharyngeal region of mice lacking Tbx1. Dev Biol 285: 554–569. 16109395 10.1016/j.ydbio.2005.06.026

[26] Liao J, Aggarwal VS, Nowotschin S, Bondarev A, Lipner S, et al (2008) Identification of downstream genetic pathways of Tbx1 in the second heart field. Dev Biol 316: 524–537. 18328475 10.1016/j.ydbio.2008.01.037 PMC2494702

[27] van Bueren KL, Papangeli I, Rochais F, Pearce K, Roberts C, et al (2010) Hes1 expression is reduced in Tbx1 null cells and is required for the development of structures affected in 22q11 deletion syndrome. Dev Biol 340: 369–380. 20122914 10.1016/j.ydbio.2010.01.020 PMC2877781

[28] Monks DC, Morrow BE (2012) Identification of putative retinoic acid target genes downstream of mesenchymal Tbx1 during inner ear development. Dev Dyn 241: 563–573. 22275070 10.1002/dvdy.23731 PMC3282991

[29] Vitelli F, Taddei I, Morishima M, Meyers EN, Lindsay EA, et al (2002) A genetic link between Tbx1 and fibroblast growth factor signaling. Development 129: 4605–4611. 12223416 10.1242/dev.129.19.4605

[30] Abu-Issa R, Smyth G, Smoak I, Yamamura K, Meyers EN (2002) Fgf8 is required for pharyngeal arch and cardiovascular development in the mouse. Development 129: 4613–4625. 12223417 10.1242/dev.129.19.4613

[31] Nowotschin S, Liao J, Gage PJ, Epstein JA, Campione M, et al (2006) Tbx1 affects asymmetric cardiac morphogenesis by regulating Pitx2 in the secondary heart field. Development 133: 1565–1573. 16556915 10.1242/dev.02309

[32] Randall V, McCue K, Roberts C, Kyriakopoulou V, Beddow S, et al (2009) Great vessel development requires biallelic expression of Chd7 and Tbx1 in pharyngeal ectoderm in mice. J Clin Invest 119: 3301–3310. 19855134 10.1172/JCI37561 PMC2769172

[33] Chen L, Mupo A, Huynh T, Cioffi S, Woods M, et al (2010) Tbx1 regulates Vegfr3 and is required for lymphatic vessel development. J Cell Biol 189: 417–424. 20439995 10.1083/jcb.200912037 PMC2867300

[34] Guo C, Sun Y, Zhou B, Adam RM, Li X, et al (2011) A Tbx1-Six1/Eya1-Fgf8 genetic pathway controls mammalian cardiovascular and craniofacial morphogenesis. J Clin Invest 121: 1585–1595. 21364285 10.1172/JCI44630 PMC3069777

[35] Voss AK, Vanyai HK, Collin C, Dixon MP, McLennan TJ, et al (2012) MOZ regulates the Tbx1 locus, and Moz mutation partially phenocopies DiGeorge syndrome. Dev Cell 23: 652–663. 22921202 10.1016/j.devcel.2012.07.010 PMC3442180

[36] Chen L, Fulcoli FG, Ferrentino R, Martucciello S, Illingworth EA, et al (2012) Transcriptional control in cardiac progenitors: Tbx1 interacts with the BAF chromatin remodeling complex and regulates Wnt5a. PLoS Genet 8: e1002571. 22438823 10.1371/journal.pgen.1002571 PMC3305383

[37] Lazebnik MB, Tussie-Luna MI, Roy AL (2008) Determination and functional analysis of the consensus binding site for TFII-I family member BEN, implicated in Williams-Beuren syndrome. J Biol Chem 283: 11078–11082. 18326499 10.1074/jbc.C800049200 PMC2431064

[38] Sidhu A, Miller PJ, Johanson KE, Hollenbach AD (2008) Novel flanking DNA sequences enhance FOXO1a DNA binding affinity but do not alter DNA bending. Biochemistry 47: 6809–6818. 18537265 10.1021/bi702495m

[39] Chen L, Zheng J, Yang N, Li H, Guo S (2011) Genomic selection identifies vertebrate transcription factor Fezf2 binding sites and target genes. J Biol Chem 286: 18641–18649. 21471212 10.1074/jbc.M111.236471 PMC3099680

[40] Djordjevic M (2007) SELEX experiments: new prospects, applications and data analysis in inferring regulatory pathways. Biomol Eng 24: 179–189. 17428731 10.1016/j.bioeng.2007.03.001

[41] Xie Q, Cvekl A (2011) The orchestration of mammalian tissue morphogenesis through a series of coherent feed-forward loops. J Biol Chem 286: 43259–43271. 21998302 10.1074/jbc.M111.264580 PMC3234836

[42] Kirstein L, Cvekl A, Chauhan BK, Tamm ER (2000) Regulation of human myocilin/TIGR gene transcription in trabecular meshwork cells and astrocytes: role of upstream stimulatory factor. Genes Cells 5: 661–676. 10947851 10.1046/j.1365-2443.2000.00355.x

[43] Chauhan BK, Yang Y, Cveklova K, Cvekl A (2004) Functional interactions between alternatively spliced forms of Pax6 in crystallin gene regulation and in haploinsufficiency. Nucleic Acids Res 32: 1696–1709. 15020706 10.1093/nar/gkh334 PMC390332

[44] Siepel A, Bejerano G, Pedersen JS, Hinrichs AS, Hou M, et al (2005) Evolutionarily conserved elements in vertebrate, insect, worm, and yeast genomes. Genome Res 15: 1034–1050. 16024819 10.1101/gr.3715005 PMC1182216

[45] Aggarwal VS, Liao J, Bondarev A, Schimmang T, Lewandoski M, et al (2006) Dissection of Tbx1 and Fgf interactions in mouse models of 22q11DS suggests functional redundancy. Hum Mol Genet 15: 3219–3228. 17000704 10.1093/hmg/ddl399

[46] Huang da W, Sherman BT, Lempicki RA (2009) Bioinformatics enrichment tools: paths toward the comprehensive functional analysis of large gene lists. Nucleic Acids Res 37: 1–13. 19033363 10.1093/nar/gkn923 PMC2615629

[47] Huang da W, Sherman BT, Lempicki RA (2009) Systematic and integrative analysis of large gene lists using DAVID bioinformatics resources. Nat Protoc 4: 44–57. 19131956 10.1038/nprot.2008.211

[48] Beermann F, Kaloulis K, Hofmann D, Murisier F, Bucher P, et al (2006) Identification of evolutionarily conserved regulatory elements in the mouse Fgf8 locus. Genesis 44: 1–6. 16397882 10.1002/gene.20177

[49] Inoue F, Parvin MS, Yamasu K (2008) Transcription of fgf8 is regulated by activating and repressive cis-elements at the midbrain-hindbrain boundary in zebrafish embryos. Dev Biol 316: 471–486. 18280464 10.1016/j.ydbio.2008.01.013

[50] Hu T, Yamagishi H, Maeda J, McAnally J, Yamagishi C, et al (2004) Tbx1 regulates fibroblast growth factors in the anterior heart field through a reinforcing autoregulatory loop involving forkhead transcription factors. Development 131: 5491–5502. 15469978 10.1242/dev.01399

[51] Simmler MC, Cohen-Salmon M, El-Amraoui A, Guillaud L, Benichou JC, et al (2000) Targeted disruption of otog results in deafness and severe imbalance. Nat Genet 24: 139–143. 10655058 10.1038/72793

[52] Kelly RG, Jerome-Majewska LA, Papaioannou VE (2004) The del22q11.2 candidate gene Tbx1 regulates branchiomeric myogenesis. Hum Mol Genet 13: 2829–2840. 15385444 10.1093/hmg/ddh304

[53] Muller CW, Herrmann BG (1997) Crystallographic structure of the T domain-DNA complex of the Brachyury transcription factor. Nature 389: 884–888. 9349824 10.1038/39929

[54] El Omari K, De Mesmaeker J, Karia D, Ginn H, Bhattacharya S, et al. (2011) Structure of the DNA-bound T-box domain of human TBX1, a transcription factor associated with the DiGeorge syndrome. Proteins. 10.1002/prot.23208 22095455

[55] Prouse MB, Campbell MM (2013) Interactions between the R2R3-MYB transcription factor, AtMYB61, and target DNA binding sites. PLoS One 8: e65132. 23741471 10.1371/journal.pone.0065132 PMC3669277

[56] Lefstin JA, Yamamoto KR (1998) Allosteric effects of DNA on transcriptional regulators. Nature 392: 885–888. 9582068 10.1038/31860

[57] Meijsing SH, Pufall MA, So AY, Bates DL, Chen L, et al (2009) DNA binding site sequence directs glucocorticoid receptor structure and activity. Science 324: 407–410. 19372434 10.1126/science.1164265 PMC2777810

[58] Lindsay EA (2001) Chromosomal microdeletions: dissecting del22q11 syndrome. Nat Rev Genet 2: 858–868. 11715041 10.1038/35098574

[59] Bussen M, Petry M, Schuster-Gossler K, Leitges M, Gossler A, et al (2004) The T-box transcription factor Tbx18 maintains the separation of anterior and posterior somite compartments. Genes Dev 18: 1209–1221. 15155583 10.1101/gad.300104 PMC415645

[60] Yasuhiko Y, Haraguchi S, Kitajima S, Takahashi Y, Kanno J, et al (2006) Tbx6-mediated Notch signaling controls somite-specific Mesp2 expression. Proc Natl Acad Sci U S A 103: 3651–3656. 16505380 10.1073/pnas.0508238103 PMC1450137

[61] Wardle FC, Papaioannou VE (2008) Teasing out T-box targets in early mesoderm. Curr Opin Genet Dev 18: 418–425. 18778771 10.1016/j.gde.2008.07.017 PMC2700021

[62] Blow MJ, McCulley DJ, Li Z, Zhang T, Akiyama JA, et al (2010) ChIP-Seq identification of weakly conserved heart enhancers. Nat Genet 42: 806–810. 20729851 10.1038/ng.650 PMC3138496

[63] McGaughey DM, Stine ZE, Huynh JL, Vinton RM, McCallion AS (2009) Asymmetrical distribution of non-conserved regulatory sequences at PHOX2B is reflected at the ENCODE loci and illuminates a possible genome-wide trend. BMC Genomics 10: 8. 19128492 10.1186/1471-2164-10-8 PMC2630312

[64] Schmidt D, Wilson MD, Ballester B, Schwalie PC, Brown GD, et al (2010) Five-vertebrate ChIP-seq reveals the evolutionary dynamics of transcription factor binding. Science 328: 1036–1040. 20378774 10.1126/science.1186176 PMC3008766

[65] Chatterjee S, Bourque G, Lufkin T (2011) Conserved and non-conserved enhancers direct tissue specific transcription in ancient germ layer specific developmental control genes. BMC Dev Biol 11: 63. 22011226 10.1186/1471-213X-11-63 PMC3210094

[66] Liao BY, Weng MP (2012) Natural selection drives rapid evolution of mouse embryonic heart enhancers. BMC Syst Biol 6 Suppl 2S1. 10.1186/1752-0509-6-S2-S1 PMC3521173 23281795

[67] Conley CA, Silburn R, Singer MA, Ralston A, Rohwer-Nutter D, et al (2000) Crossveinless 2 contains cysteine-rich domains and is required for high levels of BMP-like activity during the formation of the cross veins in Drosophila. Development 127: 3947–3959. 10952893 10.1242/dev.127.18.3947

[68] Lin J, Patel SR, Cheng X, Cho EA, Levitan I, et al (2005) Kielin/chordin-like protein, a novel enhancer of BMP signaling, attenuates renal fibrotic disease. Nat Med 11: 387–393. 15793581 10.1038/nm1217

[69] Binnerts ME, Wen X, Cante-Barrett K, Bright J, Chen HT, et al (2004) Human Crossveinless-2 is a novel inhibitor of bone morphogenetic proteins. Biochem Biophys Res Commun 315: 272–280. 14766204 10.1016/j.bbrc.2004.01.048

[70] Berkes CA, Tapscott SJ (2005) MyoD and the transcriptional control of myogenesis. Semin Cell Dev Biol 16: 585–595. 16099183 10.1016/j.semcdb.2005.07.006

[71] Borycki AG, Emerson CP (1997) Muscle determination: another key player in myogenesis? Curr Biol 7: R620–623. 9368741 10.1016/s0960-9822(06)00317-4

[72] Grifone R, Jarry T, Dandonneau M, Grenier J, Duprez D, et al (2008) Properties of branchiomeric and somite-derived muscle development in Tbx1 mutant embryos. Dev Dyn 237: 3071–3078. 18816853 10.1002/dvdy.21718

[73] El-Amraoui A, Cohen-Salmon M, Petit C, Simmler MC (2001) Spatiotemporal expression of otogelin in the developing and adult mouse inner ear. Hear Res 158: 151–159. 11506947 10.1016/s0378-5955(01)00312-4

[74] Schraders M, Ruiz-Palmero L, Kalay E, Oostrik J, del Castillo FJ, et al (2012) Mutations of the gene encoding otogelin are a cause of autosomal-recessive nonsyndromic moderate hearing impairment. Am J Hum Genet 91: 883–889. 23122587 10.1016/j.ajhg.2012.09.012 PMC3487128

[75] Birnbaum RY, Everman DB, Murphy KK, Gurrieri F, Schwartz CE, et al (2012) Functional characterization of tissue-specific enhancers in the DLX5/6 locus. Hum Mol Genet 21: 4930–4938. 22914741 10.1093/hmg/dds336 PMC3529576

