# Supplementary figures and images for: Mammalian TBX1 Preferentially Binds and Regulates Downstream Targets Via a Tandem T-site Repeat
Source: PLoS One. 2014 May 5;9(5):e95151. doi: 10.1371/journal.pone.0095151 (PMC4010391; doi:10.1371/journal.pone.0095151)

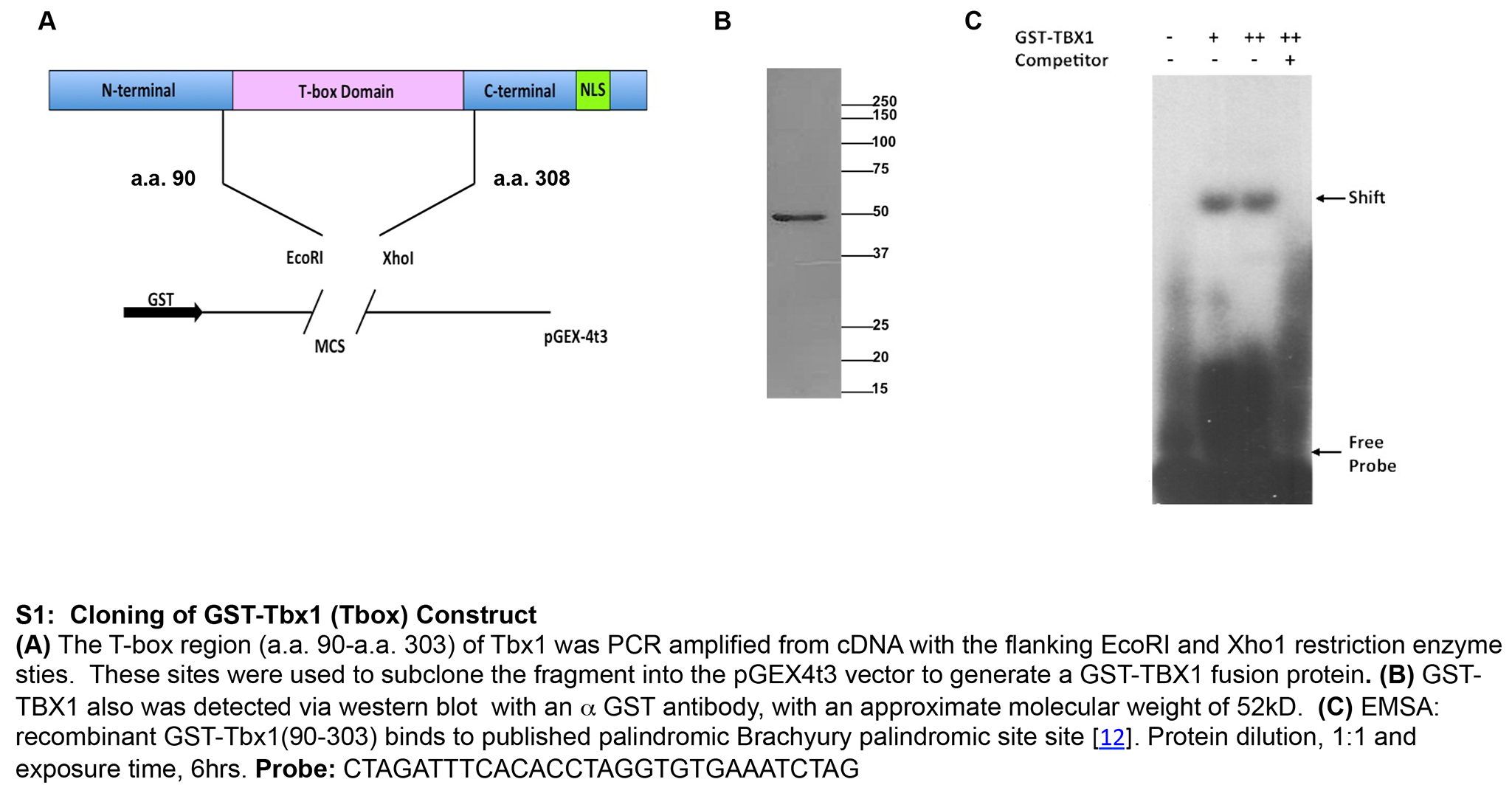

Supplement: Figure S1 — Cloning of GST-Tbx1 (T-box) Construct. A: The T-box region (amino acids 90–303) of mouse Tbx1 was PCR amplified from cDNA with flanking EcoRI and XhoI restriction enzyme sties. These sites were used to subclone the fragment into the pGEX4t3 vector (GE Healthcare) to generate a GST-TBX1 fusion protein. B: GST-TBX1 was detected via western blot with an α GST antibody, with an approximate molecular weight of 52 kD. C: EMSA with recombinant GST-TBX1 (90–303) binds to published palindromic Brachyury palindrome motif [12]. Protein dilution, 1∶1 and exposure time, 6 hrs. Probe: CTAGATTTCACACCTAGGTGTGAAATCTAG. (TIF) [file pone.0095151.s001.tif]

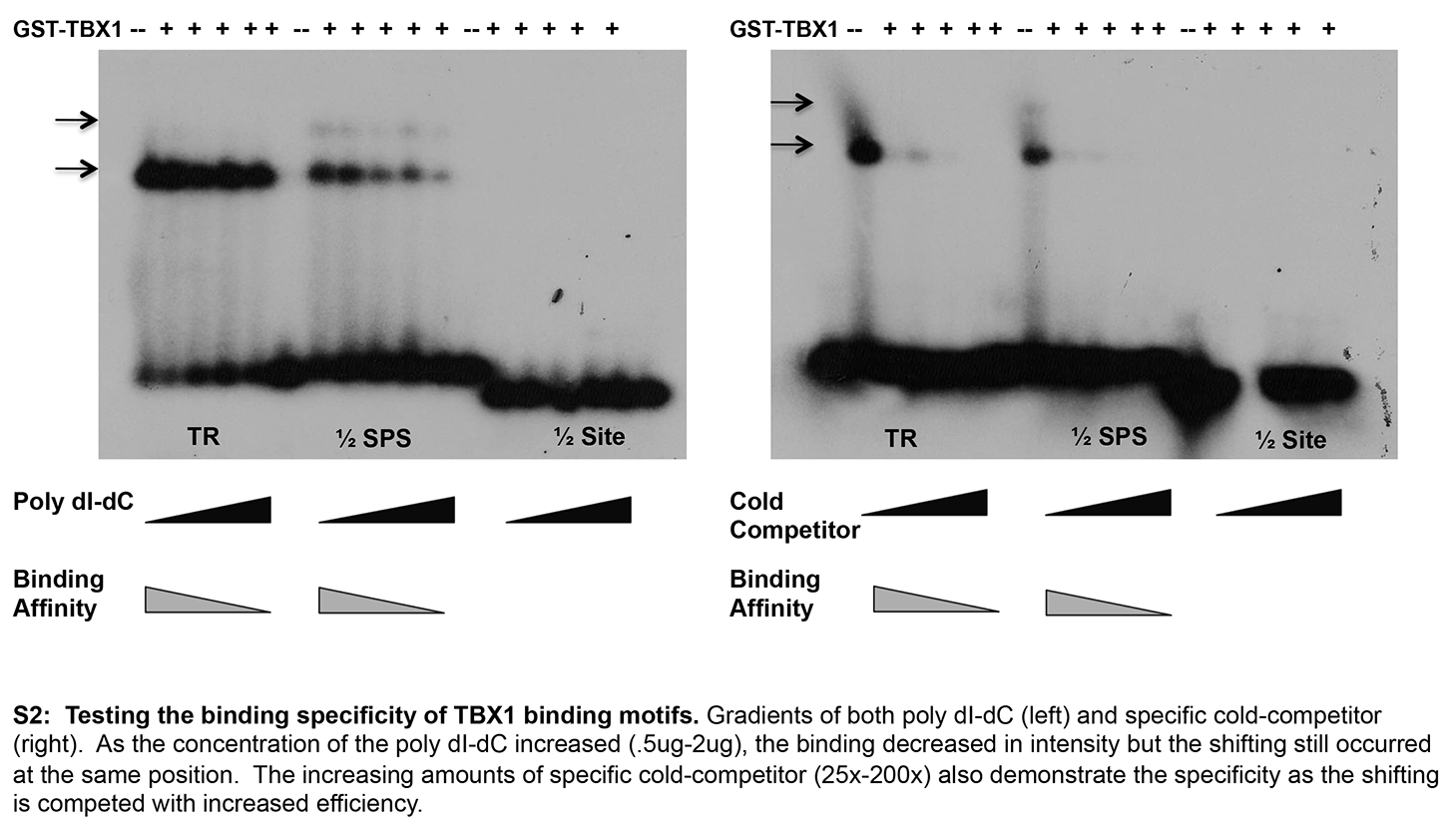

Supplement: Figure S2 — Testing the binding specificity of TBX1 binding motifs. Gradients of both poly dI-dC (left) and specific cold-competitor (right). As the concentration of the poly dI-dC increased (0.5–2 µg), the binding decreased in intensity but the creation of protein-DNA complexes still occurred at the same position. Increasing amounts of specific cold-competitor (25x-200x) was used to demonstrate the specificity of binding. (TIF) [file pone.0095151.s002.tif]

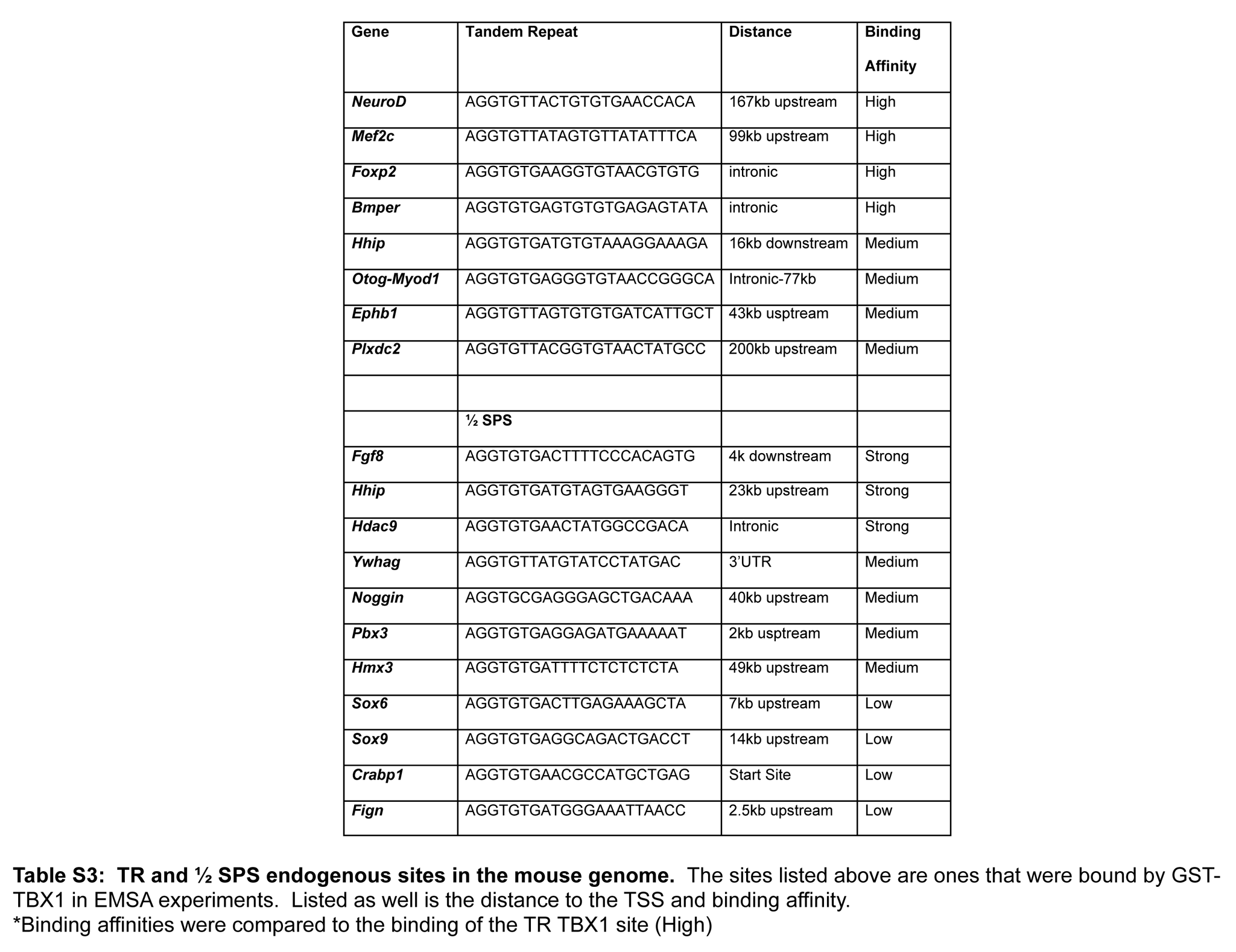

Supplement: Table S3 — TR and ½ SPS endogenous sites in the mouse genome. The sites listed above are the endogenous sites that were bound by GST-TBX1 in EMSA experiments. Listed as well is the distance to the TSS and comparative gel shift band intensity. *Gel shift bands were compared to the binding of TBX1 to the TR TBX1 site (High). (TIF) [file pone.0095151.s005.tif]
